# The growth of plants and indigenous bacterial community were significantly affected by cadmium contamination in soil–plant system

**DOI:** 10.1186/s13568-021-01264-y

**Published:** 2021-07-10

**Authors:** Yunyan Du, Dawei Zhang, Dinggang Zhou, Lili Liu, Jinfeng Wu, Hongsong Chen, Decai Jin, Mingli Yan

**Affiliations:** 1 School of Life Sciences, Hunan University of Science and Technology, Xiangtan, 411201 People’s Republic of China; 2 Key Laboratory of Environmental Biotechnology, Research Center for Eco-Environmental Sciences, Chinese Academy of Sciences, Beijing, 100085 People’s Republic of China; 3 Hunan Key Laboratory of Economic Crops Genetic Improvement and Integrated Utilization, Xiangtan, 411201 People’s Republic of China; 4 Guangxi Key Laboratory of Biology for Crop Diseases and Insect Pests, Plant Protection Research Institute, Guangxi Academy of Agricultural Sciences, Nanning, 530007 People’s Republic of China

**Keywords:** Cd, Bacterial community, Oilseed rape, Soil, Phyllosphere, Endophyte

## Abstract

Concentrations of heavy metals continue to increase in soil environments as a result of both anthropogenic activities and natural processes. Cadmium (Cd) is one of the most toxic heavy metals and poses health risks to both humans and the ecosystem. Herein, we explore the impacts of Cd on a soil–plant system composed of oilseed rapes (*Brassica napus* and *Brassica juncea*) and bacteria. The results showed that Cd accumulation within tissues of two species of oilseed rapes enhanced with increasing concentrations of Cd in soils, and Cd treatment decreased their chlorophyll content and suppressed rapeseeds growth. Meanwhile, Cd stress induced the changes of antioxidative enzymes activities of both *B. napus* and *B. juncea*. Response to Cd of bacterial community was similar in soil-two species of oilseed rapes system. The impact of Cd on the bacterial communities of soils was greater than bacterial communities of plants (phyllosphere and endophyte). The α-diversity of bacterial community in soils declined significantly under higher Cd concentration (30 mg/kg). In addition, soil bacterial communities composition and structure were altered in the presence of higher Cd concentration. Meanwhile, the bacterial communities of bulk soils were significantly correlated with Cd, while the variation of rhizosphere soils bacterial communities were markedly correlated with Cd and other environmental factors of both soils and plants. These results suggested that Cd could affect both the growth of plants and the indigenous bacterial community in soil–plant system, which might further change ecosystem functions in soils.

**Supplementary Information:**

The online version contains supplementary material available at 10.1186/s13568-021-01264-y.

## Introduction

Heavy metals (HMs) in soils have become a serious environmental issue due to their poisonousness and bioaccumulation within the food chain. They can deteriorate soil quality, reduce food safety, and threaten human health (Li et al. 2014). Moreover, these metals are not degraded by chemical or biological methods and are persistent in soil (Cheraghi-Aliakbari et al. 2020). Cadmium (Cd), one of the most toxic heavy metals, and nonessential for humans and animals, is widely present in soil due to anthropogenic activities such as smelting, mining and battery disposal (DalCorso et al. 2019; Mitra et al. 2018). Cd accumulation in food could cause serious health problems in humans and animals (Khan et al. 2017). Phytoremediation is an efficient and environmental-friendly method to remove Cd from soil and could be used to remediate Cd pollution soil (Liu et al. 2020).

Under Cd stress, plants are damaged by photosynthesis inhibition and disruption of nutrition absorption (Li et al. 2018), affecting the growth of sensitive plants (Wang et al. 2020a). However, Cd hyperaccumulators have higher tolerance to Cd, these include many types of *Brassica* species that can be grown under heavy metal stress in soil, with the whole plant being used for biofuel production (Rizwan et al. 2018). *Brassica napus* and *Brassica juncea* are widely grown around the world and have been investigated extensively for the remediation of Cd (Goswami and Das 2015; Rossi et al. 2002).

Soil is the main medium for terrestrial ecosystems, supporting productive activities, regulating nutrient flow, and maintaining ecosystem health with microbes playing a significant role in these processes (Xia et al. 2018). However, many studies have found that microbial diversity, abundance, and composition in soils are strongly affected by Cd contamination (Hou et al. 2018; Wood et al. 2016) As an essential part of ecosystems, microbial communities play an important role in global biogeochemical cycle (Beattie et al. 2018). Moreover, microorganisms are essential in biogeochemical cycling of HMs (Jing and Kjellerup 2018), with their responses to pollution having profound ecological effects and can serve as biological indicators of heavy metals such as Cd toxicity. Microorganisms are an important component of phytoremediation technology (Zhang et al. 2012). Phyllosphere (Jia et al. 2018) and endophytic bacteria (Wang et al. 2020b) are also beneficial in phytoremediation. However, less attention has been paid to the effect of Cd on plant microbial community (phyllospheric and endophytic bacterial communities) in the soil–plant ecosystem.

In this study, we chose two species of oilseed rapes (*B. napus* and *B. juncea*) to investigate the effect of Cd on plants and compared their Cd-tolerance. A 16S rRNA gene amplicon Illumina Miseq approach was performed to examine effects of Cd contamination on bacterial communities in the soil–plant ecosystem. Our results provide a detailed understanding of the effects of Cd on plants and soil–plant system microbial communities and help improve phytoremediation systems.

## Material and methods

### Greenhouse experiments

The test soil was agricultural topsoil (0–20 cm) taken from a suburb of Hunan province (27°54′15″ N, 112°55′06″ E) and the main properties of the soil prior to any treatment were as follows: pH 5.56, total organic carbon (TOC) 1.62%, total nitrogen (TN) 1803.74 mg/kg, total phosphorus (TP) 921.04 mg/kg, available phosphorus (AP) 78.14 mg/kg, available potassium (AK) 135.41 mg/kg, ammonia nitrogen (NH_3_-N) 17.65 mg/kg and nitrate nitrogen (NO_3_-N) 19.44 mg/kg and a background Cd concentration of 0 mg/kg.

The *B. napus* L. cultivar “Zhong-shuang 11” and *B. juncea* L. cultivar “Purple Leaf Mustard” was used in this experiment. The plants were grown in a greenhouse located at the Hunan University of Science and Technology (27°54′15″ N, 112°55′06″ E, Hunan, China) between October 11, 2018 and November 30, 2018. Soils were disposed by air-dried, ground and sieved by a 5 mm mesh. Then Cd aqueous solution (CdCl_2_·2.5H_2_O) was carefully added into the soil to obtain three Cd concentrations (Control (CK), 0 mg/kg Cd; concentration 1 (C10), 10 mg/kg Cd; and concentration 2 (C30), 30 mg/kg) were applied. After mixing and being allowed to stabilize for three weeks, the soil (7 kg) was transferred into plastic pots (49 cm length × 14 cm wide × 20 cm high). Seeds were sown directly into pots and ten plants were maintained in each pot finally, they were planted in the greenhouse at 20 °C ± 10 °C with suitable humidity. Every treatment was set to six replicates. Plant height and fresh weight were calculated manually and the total leaf area was measured using Image J software.

### Sample collection

Soil samples were divided into two parts, one for measuring soil physico-chemical properties while the other for conducting molecular tests. Plant samples were obtained 50 days after planting, the aboveground and belowground parts were harvested dividually by sterile scissors. Microorganisms in the soil–plant ecosystems (including the rhizosphere, bulk soil, phyllospheric, and endophytic bacterial communities) were collected on the basis of Kong et al. (2018).

### Measure of Cd content in soil and plant tissues

The samples were oven-dried, then ground and finally digested using HNO_3_ in a microwave (PyNN 140899, Peian, Beijing, China), and the contents of Cd were measured using flame atomic absorption spectrophotometer (Agilent 200 AA, Agilent Technology Co. LTD).

The Bioaccumulation Factor (BAF) and Translocation Factor (TF) of *B. napus* and *B. juncea* were calculated as follows:

$${\text{BAF}} = \frac{{\text{Content of Cd in shoots or roots (mg/kg)}}}{{\text{Content of Cd in soil (mg/kg)}}},$$

$${\text{TF}} = \frac{{\text{Content of Cd in shoots (mg/kg)}}}{{\text{Content of Cd in roots (mg/kg)}}}.$$

### Physiological index of plant tissues

The content of soluble sugar was determined by the anthrone colorimetry method (Dubois et al. 1956), using the assay kit provided by Nanjing Jiangcheng Bioengineering Institute. According to Li et al. (2013), the homogenate was collected for antioxidant enzyme activity measurement with some modifications. Commercially available assay kits (Nanjing Jiangcheng Bioengineering Institute) were used to determine the activities of superoxide dismutase (SOD, hydroxylamine method), catalase (CAT, visible light method), and peroxidase (POD, colorimetric method) according to the manufacturer’s instructions. The content of soluble protein and chlorophyll in leaf samples was measured by the Coomassie Brilliant Blue G250 staining method (Bradford 1976) and acetone extraction method (Kim et al. 2017), respectively.

### Physicochemical property of soils

Soil pH and the content of TN, TP, TOC, NH_3_-N, NO_3_-N, AP and AK were measured according to Kong et al. (2018).

### DNA extraction, PCR amplification, and sequencing

A total of 180 samples (divided equally into five parts including phyllosphere, leaf endophyte, root endophyte, rhizosphere soil and bulk soil samples) were sequenced by the following steps. DNA was extracted using Fast DNA spin kit for soil (MP Biomedicals LLC, USA). Primer 799F (5′-AACMGGATTAGATACCCKG-3′), which excludes contamination from chloroplast DNA (Beckers et al. 2016; Ghyselinck et al. 2013) and a primer designed for this study, 1115R (5′-AGGGTTGCGCTCGTTG-3′), were used to amplify V5-V6 region of the 16S rRNA gene.

The PCR reaction system (50 μL): 37.5 µl of ddH_2_O, 5 μL of 10× PCR buffer, 4 μL of 2.5 mmol/L dNTPs, 0.5 μL of 5 U/μL *Taq* DNA polymerase (TaKaRa Biotech, Beijing, China), 1 μL of 10 μmol/L forward and reverse primers and 1 μL of the DNA template (20–30 ng/μL). Cycling conditions were set as follows: 94 °C for 1 min, followed by 30 cycles of 94 °C for 20 s, 57 °C for 25 s, 72 °C for 45 s, then a final extension cycle at 72 °C for 10 min. PCR products were recovered by agarose gel electrophoresis and purified using E.Z.N.A. TM Gel Extraction Kit (Omega Biotek, Norcross, GA, USA). The purified PCR products were quantified by Nanodrop Spectrophotometer (ND-2000 Spectrophotometer, Wilmington, DE, USA). Each PCR purified product (150 ng) was mixed to construct the sequencing library. The samples were sequenced by Illumina Miseq platform at BeiJing Fixgene Co., Ltd.

### Quantitative PCR

For determining abundances of bacteria, Quantitative PCR (qPCR) was performed by using the primer pair 799F (5′-AACMGGATTAGATACCCKG-3′) and 1115R (5′-AG GGTTGCGCTCGTTG-3′) together with a CFX Connect TM Real-Time PCR Detection System (BioRad). The qPCR reaction mixture which used MonAmp™ SYBR^®^ qPCR Mix (Monad Biotech Co., Ltd) was done in a volume of 20 µl consisting of 10 μL of qPCR Mix, 1 μL of each primer, 1 μL of template DNA and 7 μL of nuclease-free water. qPCR procedure was set as follows: 95 °C for 30 s, 40 cycles of 95 °C for 5 s, 57 °C for 10 s, 72 °C for 30 s. Every sample was amplified in triplicate.

### Sequencing data process

Processing of the raw sequencing data was performed on a Galaxy pipeline (http://mem.rcees.ac.cn:8080) at Research Center for Eco-Environmental Science, China Academy of Sciences. The procedures were as follows: raw reads were assigned to different samples using barcodes, followed by removal of primers sequences, forward and reverse sequences were combined by FLASH (Magoč and Salzberg 2011), and sequences shorter than 200 bp were removed using Btrim (Kong 2011). Extraction of FASTA data from FASTQ data, checking for and removal of chimeras, and assignment of sequences with 97% identity to the same operational taxonomic unit (OTU) were performed using the UPARSE algorithm (Edgar 2013). The RDP Classifier database was used to Taxonomic assignment (Wang et al. 2007). In order to avoid the effects caused by different sequencing depth, the data was resampled randomly with the minimum number of sequences (30,000). The following analysis used the resampled OTU table.

The raw reads generated in this study have been deposited in the NCBI sequence Read Archive (Accession No. SRP283176).

### Statistical analysis

The relative abundance of top 10 phyla and top 50 genera were selected in line with the results of phyllospheric, leaf and root endophytic, rhizosphere and bulk soil bacterial communities species annotations respectively. One-way ANOVA method was conducted to analyze the significance difference (*P* < 0.05) between treatments. The relationship between environmental factors and α-diversities was investigated by pearson's correlation analysis. ANOVA method and Pearson's correlation test were performed using SPSS 21 software. The dissimilarity test was performed to evaluate the significance of clustering. Weighted principal coordinate analysis (PCoA) on the UniFrac matrix was applied to compare the different samples' bacterial community structure. Mantel test, canonical correspondence analysis (CCA) was used to show which environmental factors significantly impact microbial community structure and CCA-based variation partitioning analysis (VPA) was used to determine the contributions of Environmental Factors (EFs) to bacterial community.

## Results

### Effect of Cd on physiological properties of oilseed rapes and Cd accumulation

**Fig. 1:**
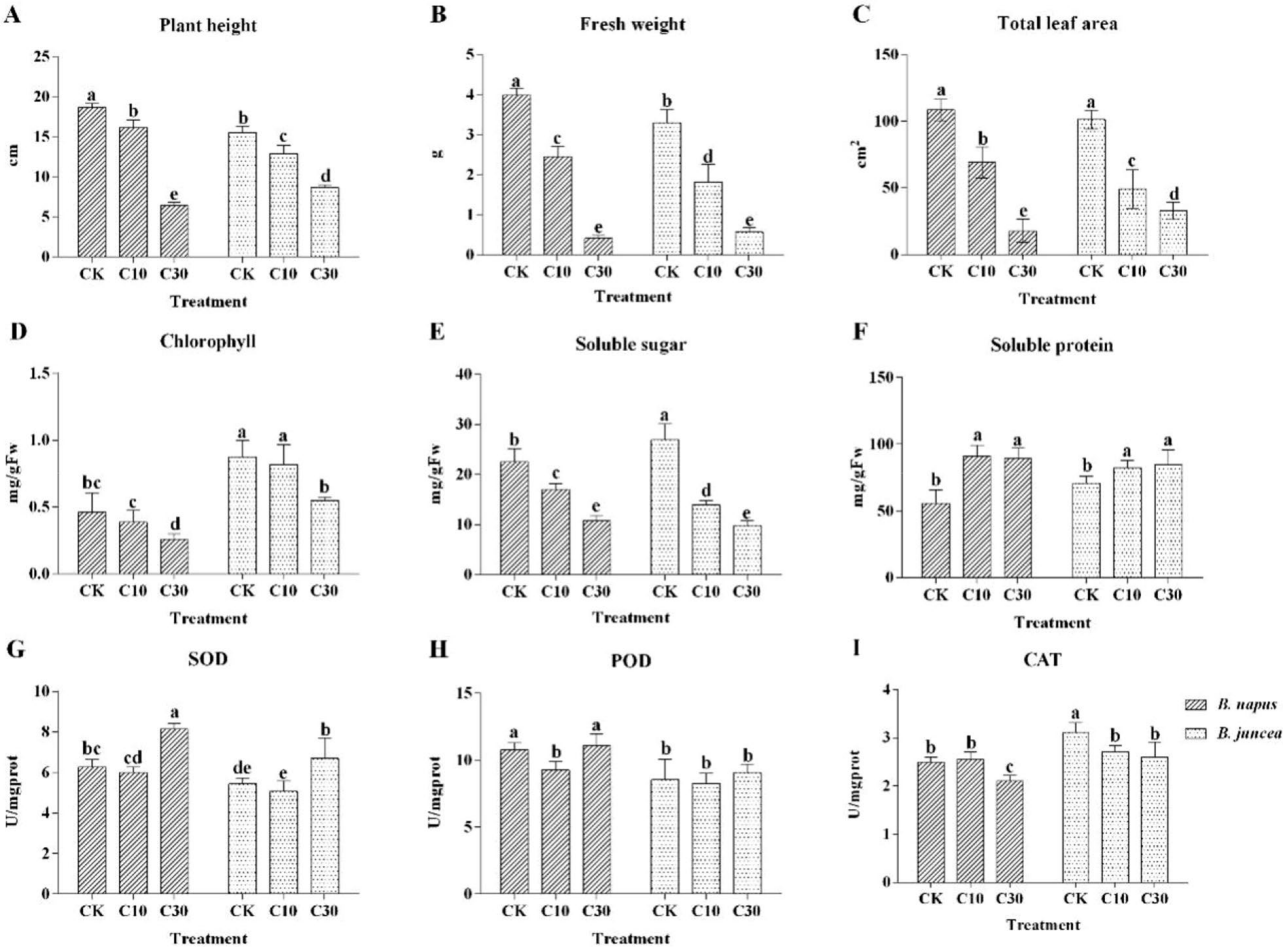
Effect of Cd on plant physiological properties. Error bars show standard deviation (n = 6). Means with different lowercase letters are significantly different at *P* < 0.05 based on one-way ANOVA. *CK* treatment with 0 mg/kg Cd, *C10* treatment with 10 mg/kg Cd, *C30* treatment with 30 mg/kg Cd, the same below

Compared to the control (CK), Cd treatment suppressed plant growth (Fig. 1), specifically plant height, fresh weight, and total leaf area were significantly reduced with increasing Cd concentration (*P* < 0.05). At CK and lower Cd concentration (10 mg/kg), the biomass of *B. napus* was significantly higher than *B. juncea,* but at higher Cd concentration (30 mg/kg) those tendencies were reversed. Pearson correlation analysis showed that Cd content in tissues was negatively correlated with plant height, weight, and leaf area in *B. napus* and *B. juncea* (*P* < 0.01) (Additional file 1: Table S1, S2)*.*

Chlorophyll was significantly decreased under higher Cd concentration, with *B. juncea* being significantly less affected than *B. napus* (*P* < 0.05) (Fig. 1D). Soluble sugar content was markedly decreased under Cd stress in both of *B. napus* and *B. juncea*, while the content of soluble protein was markedly increased under Cd treatment when compared with CK (*P* < 0.05) (Fig. 1E, F). SOD and POD activity values showed similar trends, decreasing at first and then increasing. The SOD activity at 30 mg/kg was significantly higher than in other treatments (*P* < 0.05). However, CAT activity was notably inhibited at the higher Cd concentration (*P* < 0.05). SOD and POD activity were higher in *B. napus* than *B. juncea*, while CAT activity values were the reverse (Fig. 1G–I). Pearson correlation test showed that plant Cd content was negatively related with chlorophyll, soluble sugar, and CAT activity (*P* < 0.01) and positively related with soluble protein and SOD activity (*P* < 0.05) in *B. napus* and *B. juncea* (Additional file 1: Table S1, S2)*.*

The concentration of Cd in oilseed rape leaves and roots were significantly higher with increasing Cd levels, and Cd content in *B. napus* tissues was significantly higher than *B. juncea* (*P* < 0.05) (Additional file 1: Figure S1). Translocation Factor (TF) in the two oilseed rapes species significantly decreased with increasing Cd and was higher in *B. napus* than *B. juncea* (*P* < 0.05) (Additional file 1: Table S3). Bioaccumulation Factor (BAF) in leaves and roots of both *B. napus* and *B. juncea* was bigger than one and higher in C30 (30 mg/kg) treatment compared to C10 (10 mg/kg) treatment, especially in roots (Additional file 1: Table S3).

### Effect of Cd on physicochemical properties of soils

**Fig. 2:**
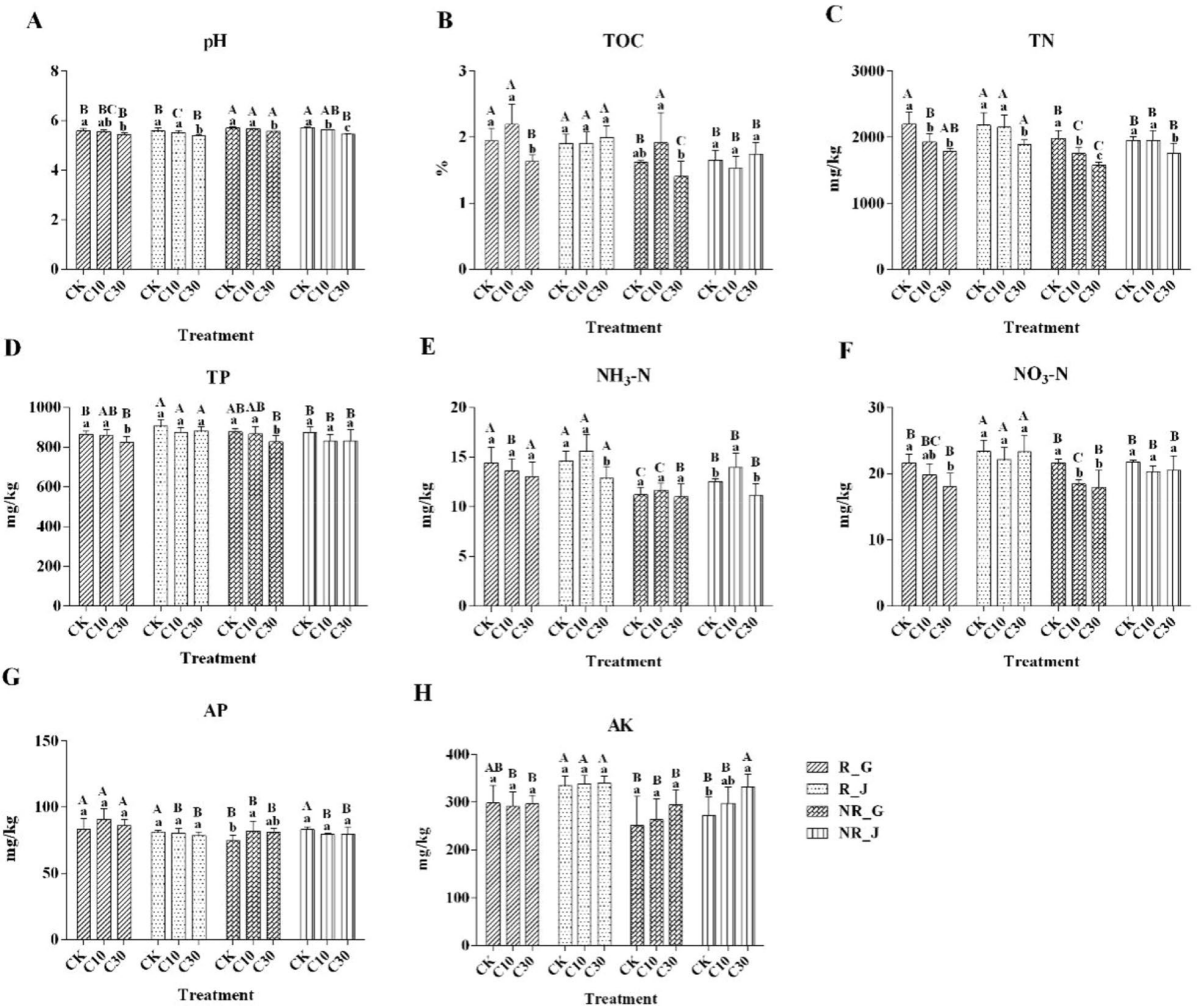
Effect of Cd on physicochemical properties of soils. Error bars show standard deviation (n = 6). Means with different lowercase letters are significantly different at *P* < 0.05 based on one-way ANOVA. *R* rhizosphere soil samples, *NR* bulk soil samples, *G* *B. napus*, *J* *B. juncea*, *CK* treatment with 0 mg/kg Cd, *C10* treatment with 10 mg/kg Cd, *C30* treatment with 30 mg/kg Cd

In *B. napus*, the pH, TN, TP, and NO_3_-N were significantly decreased under the higher Cd concentration (*P* < 0.05) and TOC first increased and then decreased in both rhizosphere and bulk soils (Fig. 2), meanwhile TOC, TN, NH_3_-N, and AP were higher, and pH lower, in rhizosphere than bulk soil. pH and TN were markedly reduced in the 30 mg/kg Cd treatment (*P* < 0.05) and NH_3_-N was first increased and then decreased in both rhizosphere and bulk soils, while AK was significantly increased (*P* < 0.05) in bulk soil of *B. juncea* under the higher Cd concentration (Fig. 2). Furthermore, TOC, TN, TP, NH_3_-N, NO_3_-N, and AK were higher, and pH was lower in rhizosphere than bulk soil in *B. juncea*. Compared with *B. napus*, most soil nutrients were higher in *B. juncea* samples under the higher Cd treatment.

Pearson correlation analysis showed that for *B. napus*, the Cd content was negatively correlated with pH, TN, TP, and NO_3_-N content in both rhizosphere and bulk soils and with TOC of the rhizosphere (Additional file 1: Table S4, S6). Cd was negatively correlated with pH, TN, and NH_3_-N content in rhizosphere and bulk soils and negatively correlated with AP of the rhizosphere, but positively correlated with AK of bulk soil in *B. juncea* (*P* < 0.05) (Additional file 1: Table S5, S7).

### Effect of Cd on bacterial numbers in soils

The results revealed that there are not significantly change of total bacterial numbers between treatment in rhizosphere, but bacterial numbers in bulk soil of *B. napus* was significantly increased under Cd stress (*P* < 0.05) (Additional file 1: Figure S2). Pearson analysis showed that Cd was positively correlation with bacterial numbers in bulk soil of *B. napus* (Additional file 1: Table S8). Meanwhile, no significant difference was observed between *B. napus* and *B. juncea* (*P* > 0.05).

### Effect of Cd on the α-diversity of bacterial community

After removing low quality reads and chimaeras in 108 plant samples and 72 soil samples, a total of 13,352,813 high-quality 16S rRNA gene reads were obtained, which were clustered into 14,359 phylotypes (OTUs) by grouping at a 97% identity threshold. The sequencing depths of all samples were appropriate for downstream analyses (Additional file 1: Figure S3).

In the plant samples, Cd mainly affected root endophytic community of *B. napus* and phyllosphere community of *B. juncea*. Shannon index and richness of *B. napus*’s root endophytes and the richness and Chao1 of *B. juncea*’s phyllosphere decreased significantly at the higher Cd concentration (*P* < 0.05) (Additional file 1: Figure S4). Pearson correlation analysis demonstrated that plant physiological factors mainly correlated with α-diversity indexes of root endophytes in *B. napus* (Additional file 1: Table S10) and that the majority of plant physiological properties were cardinally correlated with OTU numbers (richness and Chao1) of phyllosphere in *B. juncea* (Additional file 1: Table S11). pH and TOC were significantly positive correlated with and Cd was significantly negative correlated with the α-diversity of root endophytic bacterial communities in *B. napus* (Additional file 1: Table S10)*.*

**Fig. 3:**
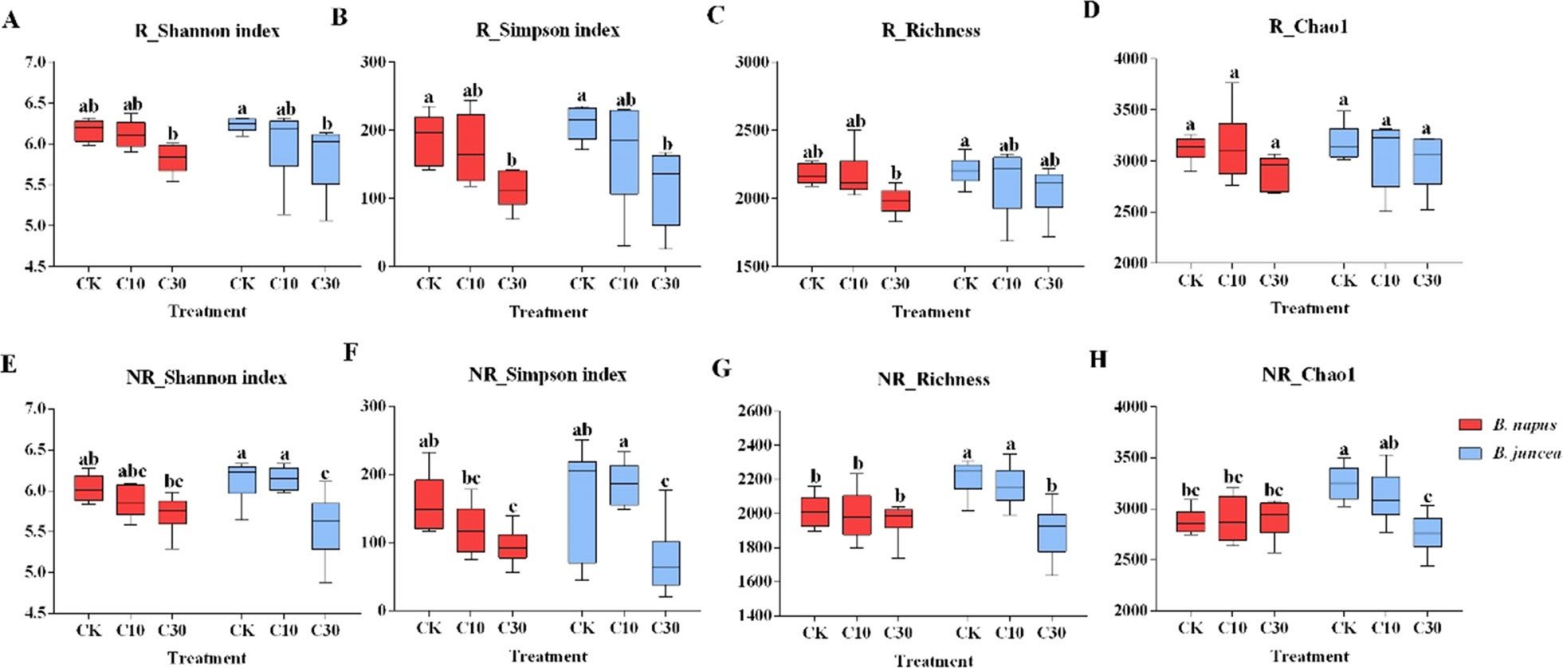
The effect of Cd on α-diversity of soil bacterial communities. **A** Shannon index; **B** Inv_Simpson index; **C** Richness; **D** Chao1 of rhizosphere bacterial communities under different cadmium treatment; **E** Shannon index; **F** Inv_Simpson index; **G** Richness; **H** Chao1 of bulk soil bacterial communities under different cadmium treatment; Means with different small letters are significantly different from one another under the different cadmium concentration treatments, and different rapeseed types (*P* < 0.05)

However, high level of Cd significantly depressed the α-diversities in the soil bacterial communities. Simpson index of rhizosphere and bulk soil was markedly reduced in the 30 mg/kg Cd treatment in *B. napus* (*P* < 0.05) (Fig. 3). Shannon and Simpson indexes of rhizosphere and Shannon, Simpson, richness and Chao1 of bulk soils in *B. juncea* were significantly decreased under the higher Cd treatment (*P* < 0.05) (Fig. 3). Most α-diversity indexes between the two species of oilseed rapes had no significant differences.

Pearson tests showed that plant’s physiological factors mainly influenced α-diversity indexes of the rhizosphere in *B. napus* and bacterial diversity (Shannon and Simpson indexes) of the rhizosphere in *B. juncea* (Additional file 1: Table S12, S13). pH, TOC, TN, TP, and NO_3_-N were positively correlated with the α-diversity in rhizosphere of *B. napus*. Meanwhile, TN and NO_3_-N were positively correlated with bacterial diversity (Shannon and Simpson indexes) of bulk soil in *B. napus* samples (Additional file 1: Table S12). In *B. juncea*, pH was positively, and AK negatively, correlated with α-diversity in bulk soil (Additional file 1: Table S13). However, Cd concentration showed a significant negative correlation with α-diversity of soil bacteria communities for both species of oilseed rapes (*P* < 0.05) (Additional file 1: Table S12, S13).

### Effect of Cd on bacterial community composition and structure

Cd could affect the composition of bacterial communities in soil–plant ecosystem, particularly under higher levels of Cd stress. In the plant bacterial community, *Proteobacteria* was dominant phylum (Additional file 1: Figure S5A). Under Cd treatment, the relative abundance of *Proteobacteria* was decreased in the *B. napus* phyllosphere, and decreased at first and then increased in the *B. napus* endophyte. However, in the *B. juncea*, the relative abundance of *Proteobacteria* of phyllosphere and root endophyte was increased and was decreased in leaf endophyte. In the soil bacterial community, *Proteobacteria* and *Actinobacteria* were dominant phyla (Additional file 1: Figure S5B). The relative abundance of *Proteobacteria* of soil bacterial community was increased under Cd stress. The relative abundance of *Actinobacteria* was decreased under higher Cd stress in the *B. napus* and was increased in the *B. juncea* under Cd treatment.

**Fig. 4:**
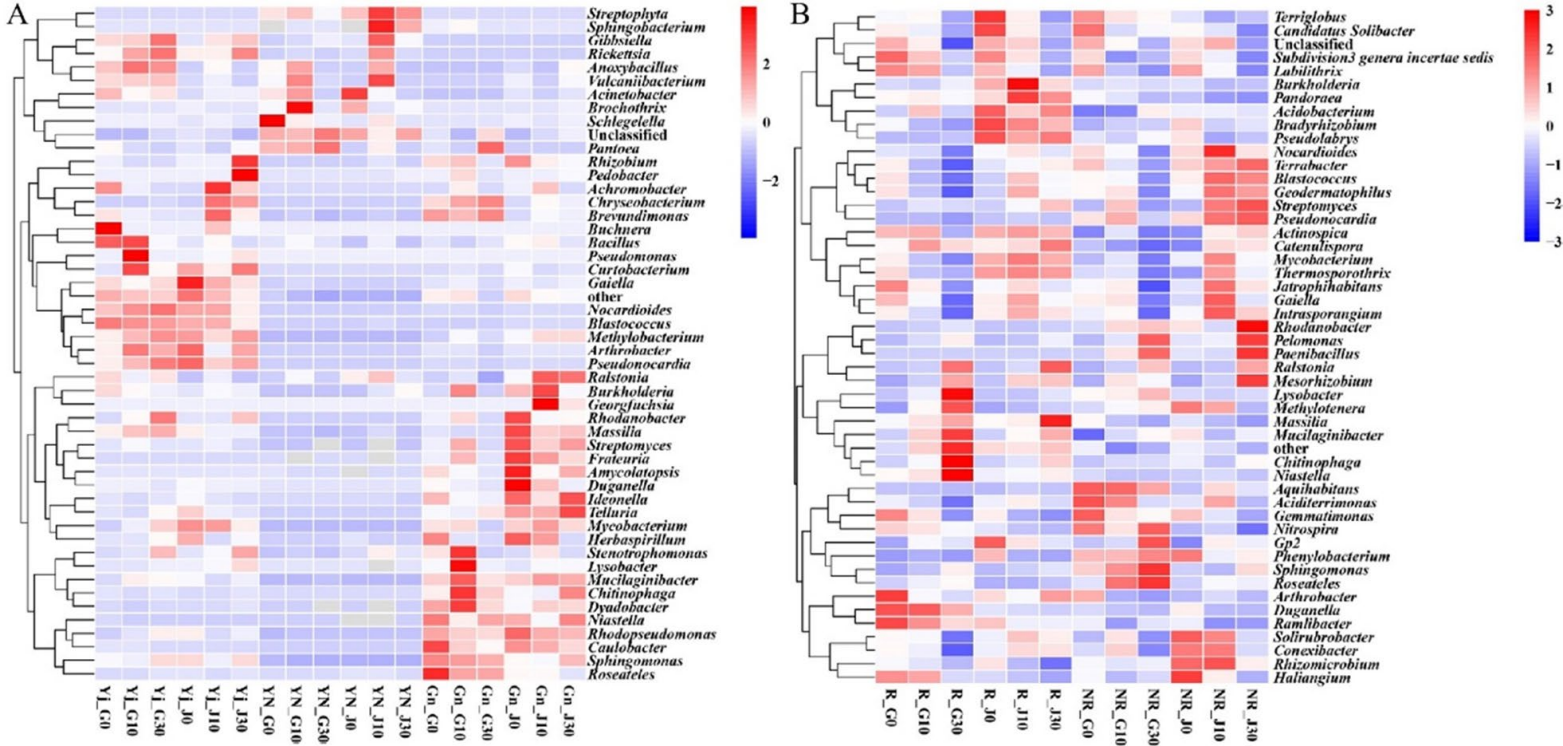
Heatmap of the relative abundance of top 50 genera in plant (**A**) and soil (**B**) samples. Abbreviations: G0/J0, treatment with 0 mg/kg Cd in *B. napus*/*B. juncea*; G10/J10, treatment with 10 mg/kg Cd in *B. napus*/*B. juncea*; G30/J30, treatment with 30 mg/kg Cd in *B. napus*/*B. juncea*; *Yj* phyllosphere samples, *Yn* endophytes samples from leaves, *Gn* endophytes samples from roots, *R* rhizosphere soil samples, *NR* bulk soil samples, the same below

At the genus level (Fig. 4), the relative abundance of some genera was altered under Cd treatment. In the *B. napus* phyllosphere samples, the relative abundances of *Massililia* sp., *Rhodanobacter* sp., and *Rickettsia* sp. were increased, and *Buchera* sp., *Achromobacter* sp., and *Acinetobacter* sp. were decreased under Cd treatment. While in *B. juncea* phyllosphere samples, *Lysobacter* sp., *Stenotrophomonas* sp., and *Gibbsiella* sp. were increased, while *Gaiell* sp., *Telluria* sp., and *Herbaspirillum* sp. were decreased under Cd treatment. In leaf endophyte samples, *Brochothrix* sp. and *Acinetobacter* sp. were increased in *B. napus* but decreased in *B. juncea* under Cd treatment. In root endophyte samples, *Chryseobacterium* sp. and *Pantoea* sp. were increased and *Caulobacter Ideonella* sp. and *Herbaspirillum* sp. were decreased in *B. napus*. *Sphingomonas* sp., *Ralstonia* sp., and *Methylobacterium* sp. were increased, and *Rhizobium* sp., *Rhodanobacter* sp., and *Duganella* sp. were decreased in *B. juncea* under Cd treatment.

In the rhizosphere, *Niastella* sp., *Methylotenera* sp., and *Lystobacter* sp. were increased and *Arthrobacter* sp., *Gemmatimanas* sp., and *Haliangium* sp. were decreased in *B. napus* under 30 mg/kg Cd treatment. *Massilia* sp., *Ralstonia* sp., and *Streptomyces* sp. were increased and *GP2* sp., *Terriglobus* sp., and *Candidatus Solibacter* sp. were decreased in *B. juncea* under 30 mg/kg Cd treatment. In bulk soil, *Sphingomonas* sp., *Rhodanobacter* sp., and *Roseateles* sp. were increased and *Arthrobacter* sp., *Gemmatimonas* sp., and *Terriglobus* sp. were decreased in *B. napus*. *Streptomyces* sp., *Pseudomocardia* sp., and *Blastococcus* sp. were increased and *Haliangium* sp., *Phenylobacterium* ap., and *Gemmatimonas* sp. were decreased in *B. juncea* under Cd treatment.

**Fig. 5:**
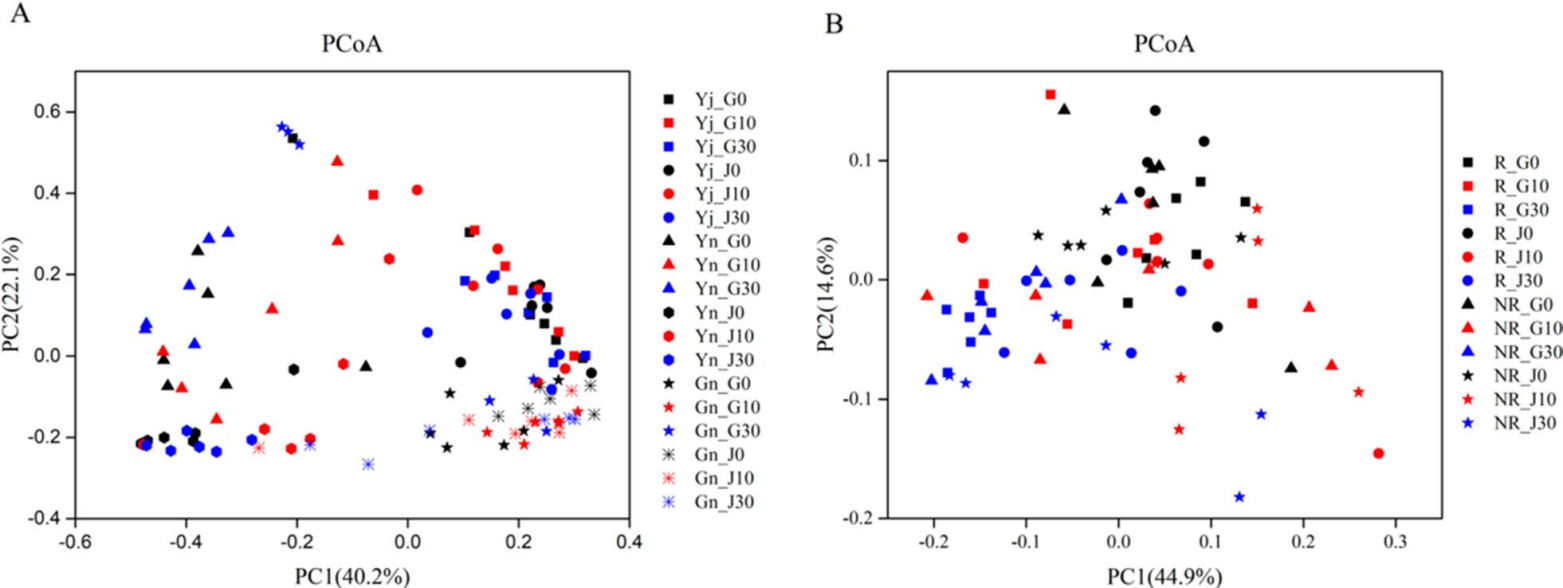
Principal coordinate analysis (PCoA) of bacteria communities in plants (**A**) and soils (**B**)

The principal co-ordinates analysis (PCoA) (Fig. 5) and dissimilarity analysis (Additional file 1: Table S14, S15) indicated that the bacterial community structures of both rhizosphere and bulk soil in both *B. napus* and *B. juncea* were significantly changed under higher Cd concentration compared to control (*P* < 0.05), but not significantly affected plant bacterial community structures.

### Relationship between microbial community structure and environmental factors

The result of Mantel test showed that there are no significant association between most environment factors and phyllosphere or leaf endophyte bacterial communities (Additional file 1: Table S16, S17). Biomass (height, weight, and leaf area), TOC and root Cd had significant association with root endophyte bacterial community in *B. napus* (Additional file 1: Table S16). Biomass, pH, NO_3_-N and Soil_Cd had significant association with rhizosphere bacterial communities and TN and Soil_Cd had significant association with bulk soil bacterial communities in *B. napus* (Table 1). Biomass, TN and Soil_Cd were significant correlated with rhizosphere bacterial community in *B. juncea* and pH and Soil_Cd were significant correlated with bulk soil bacterial community in *B. juncea* (Additional file 1: Table S18).

**Table 1:** Mantel analysis of the relationship between the soil bacterial community structure in *B. napus* and environmental factors based on Bray Curtis (BC) and Jaccard (JC) method

Samples	Environmental factors	*r*.BC	*p*.BC	*r*.JC	*p*.JC
Rhizosphere soil	Height	0.6394	0.001	0.3089	0.001
	Weight	0.5874	0.001	0.2704	0.001
	Leaf_area	0.5709	0.001	0.2964	0.001
	pH	0.2294	0.031	0.0347	0.348
	TOC	0.1250	0.192	0.0323	0.336
	TN	0.1993	0.056	0.0037	0.421
	TP	0.0518	0.293	− 0.0074	0.475
	NH_3_-N	0.1902	0.075	− 0.0443	0.567
	NO_3_-N	0.2159	0.028	− 0.0811	0.711
	AP	− 0.1334	0.899	0.2172	0.081
	AK	− 0.0977	0.777	− 0.1445	0.804
	Soil_Cd	0.6319	0.001	0.3198	0.003
Bulk soil	pH	0.1710	0.092	0.0154	0.412
	TOC	0.0624	0.361	0.0554	0.326
	TN	0.2064	0.035	0.1129	0.158
	TP	− 0.0074	0.507	− 0.0625	0.631
	NH_3_-N	− 0.1643	0.886	− 0.0580	0.608
	NO_3_-N	0.0963	0.143	0.0007	0.482
	AP	0.1708	0.14	− 0.0058	0.481
	AK	0.1677	0.108	− 0.0081	0.481
	Soil_Cd	0.2343	0.018	0.0764	0.243

**Fig. 6:**
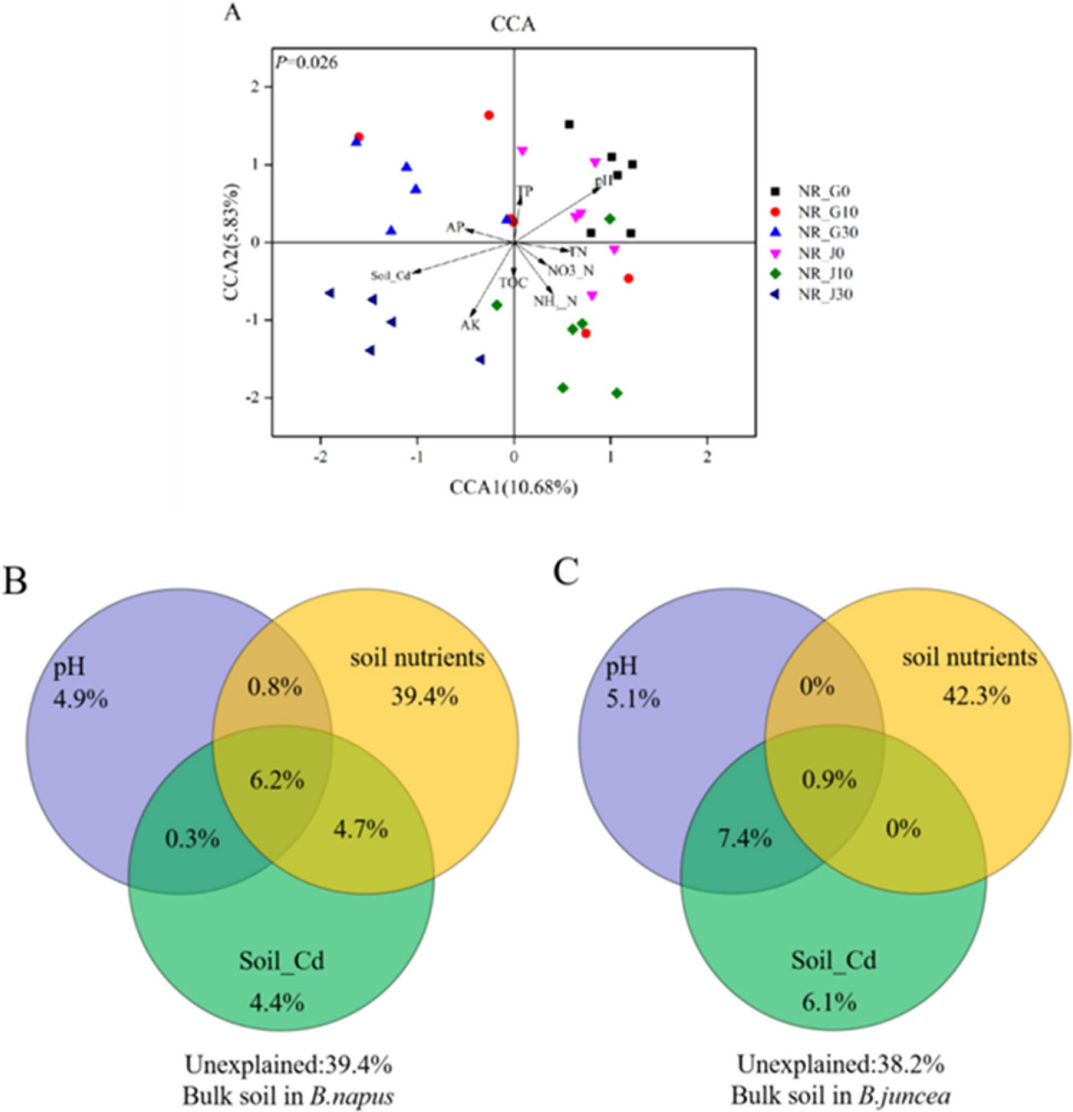
Canonical correspondence analysis (CCA) on bulk soil bacterial communities with the environmental variables (**A**) and CCA-based variation partitioning analysis (VPA) of bacterial communities explained by environmental variables (**B**, **C**)

The CCA model of root endophyte, rhizosphere and bulk soil bacterial community were significant (*P* < 0.05, Additional file 1: Figure S6A, S7A and Fig. 6A). The results of VPA indicated that biomass, pH, soil nutrients, and Root_Cd explained 14.3%, 5.4%, 37.4%, and 7.2% of variation in *B. napus* (Additional file 1: Figure S6B) and 15.5%, 6.9%, 40.2%, and 4.2% of variation in *B. juncea* (Additional file 1: Figure S6C) in root endophyte bacterial community, respectively.

CCA-based VPA indicated that biomass, pH, soil nutrients, and Cd concentration of rhizosphere soil bacterial community explained 13.8%, 4.7%, 32.6%, and 3.6% of variation in *B. napus* (Additional file 1: Figure S7B), and 14.7%, 3.4%, 35.7%, and 5.3% of variation in *B. juncea* (Additional file 1: Figure S7C), respectively. For bulk soil bacterial community, VPA indicated that pH, soil nutrients, and Cd explained 4.9%, 39.4%, and 4.4% variation in *B. napus* (Fig. 6B) and 5.1%, 42.3%, and 6.1% variation in *B. juncea* (Fig. 6C), respectively.

## Discussion

In this research, we comprehensively studied the effect of Cd contamination on soil–plant system by comparing the variance of bacterial community under different Cd treatments. The results reflected that Cd could inhibit the growth of both species of oilseed rapes, and change the bacterial community structure in soil–plant ecosystem, especially in soil.

Cd could be accumulated in plants from soil via root absorption (Khan, et al. 2017). In the current study, Cd content in *B. napus* and *B. juncea* tissues both increased with increasing Cd levels (Additional file 1: Figure S1). Hyperaccumulators generally have BAF values higher than 1 (Gascó et al. 2019). The results revealed that BAF in both *B. napus* and *B. juncea*, leaves and roots, were higher than 1 indicating that they are accumulator plants (shoots and roots) for Cd (Additional file 1: Table S3). TF in two oilseed rapes species was significantly decreased with elevated levels of Cd, which indicated the translocation capability of rapeseeds decreased under higher Cd treatment (Additional file 1: Table S3). Zeng et al. (2020) also found that TF values were decreased when the Cd concentration was elevated. It should be noted that Cd accumulation in tissues and TF values in *B. napus* were significantly higher than *B. juncea*. It may be due to different adsorption coefficients of Cd by different plant (Rattan et al. 2005).

Additionally, the accumulation of Cd in plants could also cause plant growth inhibition (Fig. 1). The biomass was declined with increasing Cd levels. This inhibition might be due to Cd inhibiting photosynthesis, retarding plant growth, since the content of chlorophyll decreased with increasing Cd concentration (Fig. 1D). One recent report demonstrated that total chlorophyll concentrations decreased with increasing Cd concentrations, which was accompanied by the reduction of biomass (Liu et al. 2011). However, in comparison between the two species of oilseed rapes, biomass of *B. juncea* was markedly higher than *B. napus* under the higher Cd concentration (30 mg/kg) while *B. napus* was significantly higher than *B. juncea* under CK treatment. The result indicated that *B. juncea* might have stronger Cd resistance than *B. napus*.

When plants experience Cd stress, antioxidative enzymes are induced (Belimov et al. 2007). In the current study, SOD and POD activities were elevated under the higher Cd concentration compared with CK, especially for SOD activity (Fig. 1G), while CAT activity was opposite (Fig. 1I). SOD is the first line to defend against ROS, so its activity increasing means that ROS production was enhanced. Increases in SOD activity could enhance H_2_O_2_ production, which is accompanying with an inhibition of CAT activity (Giansoldati et al. 2012). In this study, SOD and POD activities were higher and CAT activity was lower in *B. napus* than *B. juncea,* which indicates Cd may cause more damage to cells in *B. napus*.

The above results revealed that *B. juncea* may have relatively higher Cd-tolerance than *B. napus*. Molnár et al. (2020) found that higher levels of zinc oxide nanoparticles (ZnO NPs) were toxic to oilseed rapes and *B. juncea* has higher tolerance to ZnO NPs than *B. napus*. This may be due to the higher chlorophyll content of *B. juncea* under all treatments (Fig. 1D).

Cd, which is extensively found in agricultural soils (Guo et al. 2017), might interfere with the metabolic activity of local resident microorganisms, resulting in modified soil conditions (Lu et al. 2013). In this study, a portion of soil physiochemical properties was altered under 30 mg/kg Cd contamination (Fig. 2). The result showed that pH was lower with the increase of Cd levels and it might be closely correlated with microbial activity (Deng et al. 2018). Nitrogen is an essential nutrient for all life forms and could limit the primary productivity in many ecosystems (Frey et al. 2004). Several types of nitrogen content (NH_3_-N, NO_3_-N, and TN) were significantly decreased under higher Cd concentration (30 mg/kg) (Fig. 2), indicating that Cd may detrimentally influence soil quality, possibly be due to a detrimental effect of the heavy metal on the nitrogen cycle. Work by Sarria Carabalí et al. (2020) showed that Cd contamination has serious effects on nitrogen mobilization.

The soil microorganism plays a role in soil ecosystem functions (Schulz et al. 2013). The α-diversity of soil bacterial communities decreased under higher Cd concentration in soils (Fig. 3). The relative abundance of *Actinobacteria* was significantly decreased and *Proteobacteria* was significantly increased in the *B. napus* rhizosphere bacterial community. This is in agreement with Wu et al. (2018) who found that *Actinobacteria* abundance was significantly decreased under Cd stress, with a increase of *Proteobacteria*.

Cd pollution could change the relative abundance on genus level (Fig. 4B) including *Sphingomonas* sp., *Lysobacter* sp., and *Ralstonia* sp. which increased with the increase of Cd levels and these genera could resist Cd pollution (Guo et al. 2017; Nies 2000; Tipayno et al. 2018). Additionally, the relative abundance of *Gemmatimonas* sp., *Arthrobacter* sp., *Haliangium* sp., and *Terriglobus* sp., decreased with increasing Cd concentration. The increase of resistant groups and the reduction of sensitive groups might cause the change (Singh et al. 2014).

Results from dissimilarity tests and PCoA indicated that the soil bacterial community structures were obviously altered under the higher Cd concentration (30 mg/kg) (Additional file 1: Table S14 and Table S15; Fig. 5B). Hou et al. (2018) found that α-diversity was significantly decreased and bacterial community structure was altered in rice when Cd levels were elevated. Wood et al. (2016) also found that Cd significantly changed bacterial community structure with no reduction in bacterial number in the Cd-accumulating plant. Results of Mantel test, CCA, and CCA-based VPA (Table 1; Fig. 6; Additional file 1: Table S18, Figure S7) suggested the bacterial community in soil samples is most significantly and negatively correlated with Cd stress, which was in accordance with previous findings (Beattie et al. 2018).

There were no significant effects to the bacterial α-diversity or community structure under 10 mg/kg Cd stress in soils, possibly due to the presence of the oilseed rapes. Wang et al. (2018) found that Cd stress reduced microbial α-diversity, while planting with oilseed rape increased the microbial α-diversity, alleviating the toxic effects of Cd in soils. The result revealed that both of *B. napus* and *B. junca* could reduce the toxicity of Cd to bacteria.

Although many studies have concerned the influence of Cd on soil microbial communities, less research has paid attention on plant microbial community (phyllospheric and endophytic) affected by contaminant. The phyllosphere and endosphere bacteria are important in remediation of heavy metal pollutions (Jia et al. 2018; Wang et al. 2020b) and some of them could promote the plant growth (Bulgarelli et al. 2013). In this study, there are almost no significant variation for plant bacterial community diversities and structure (phyllospheric and endophytic bacterial community) under Cd stress (Additional file 1: Figure S4), but Cd contamination could change the relative abundance of some genera (Fig. 4A). Some of these bacteria had reported that are beneficial for soil–plant ecosystems. For example, *Ralstonia* sp. and *Methylobacterium* sp. were Cd-resistant bacteria (Lodewyckx et al. 2002; Nies 2000). The variation of the above bacteria under Cd imposition may lead to the change of the ecological function in the soil–plant ecosystem.

This study demonstrated the effect of Cd on the soil–plant ecosystem. Cd suppressed the growth of oilseed rapes (*B. napus* and *B. juncea*) and the physiological traits of plants were changed under Cd stress. Meanwhile, Cd primarily affected pH and nitrogen in soil physicochemical properties. Accumulation of Cd in plants increased and TF was decreased with increasing Cd level in soils, and both *B. napus* and *B. juncea* were accumulators for Cd because of BAF higher than 1. The bacterial communities in soil were significantly altered under higher Cd treatments while no significant difference was found for plant bacterial community. Additionally, *B. juncea* may have stronger Cd tolerance than *B. napus* under higher Cd concentration. This research offers a new perspective for the impact of contaminants on soil–plant system and might be help improve phytoremediation efficiency.

## Supplementary Information

**Additional file1. **Additional data.

## Data Availability

The authors declare that all data obtained have been included into the manuscript and its additional files.
