# Supplementary material for: The growth of plants and indigenous bacterial community were significantly affected by cadmium contamination in soil–plant system
Source: AMB Express. 2021 Jul 10;11:103. doi: 10.1186/s13568-021-01264-y (PMC8272791; doi:10.1186/s13568-021-01264-y)
Supplement: Supplementary file 1 — Additional file1. Additional data. [file 13568_2021_1264_MOESM1_ESM.docx]

**Additional file 1.** Additional data

**Table S1 Pearson correlation of Cd concentration in plants of *B. napus* with plant physiological properties.** Note: A: Negative correlation; ns: Not significant; *: Correlation is significant at the 0.05 level (2-tailed); **: Correlation is significant at the 0.01 level (2-tailed), the same below.

| Pearson correlation | Leaf_Cd | Root_Cd | Height | Weight | | Leaf_area | Sugar | Protein | chlorophyll | SOD | POD | CAT |
| --- | --- | --- | --- | --- | --- | --- | --- | --- | --- | --- | --- | --- |
| Leaf_Cd  Root_Cd | 1 |  |  | |  |  |  |  |  |  |  |  |
| Root_Cd | ** | 1 |  | |  |  |  |  |  |  |  |  |
| Height | A** | A** | 1 | |  |  |  |  |  |  |  |  |
| Weight | A** | A** | ** | | 1 |  |  |  |  |  |  |  |
| Leaf_area | A** | A** | ** | | ** | 1 |  |  |  |  |  |  |
| Sugar | A** | A** | ** | | ** | ** | 1 |  |  |  |  |  |
| Protein | ** | * | A* | | A** | A** | A** | 1 |  |  |  |  |
| Chlorophyll | A** | A** | ** | | ** | ** | * | ns | 1 |  |  |  |
| SOD | ** | ** | A** | | A** | A** | A** | ns | A* | 1 |  |  |
| POD | ns | ns | ns | | ns | ns | ns | ns | ns | ** | 1 |  |
| CAT | A** | A** | ** | | ** | ** | ** | ns | ** | A** | A* | 1 |

**Table S2 Pearson correlation of Cd concentration in plants of *B. juncea* with plant physiological properties.**

| Pearson correlation | Leaf_Cd | Root_Cd | Height | Weight | Leaf_area | Sugar | Protein | Chlorophyll | SOD | POD | CAT |  |
| --- | --- | --- | --- | --- | --- | --- | --- | --- | --- | --- | --- | --- |
| Leaf_Cd | | 1 |  |  |  |  |  |  |  |  |  |  |
| Root_Cd | | ** | 1 |  |  |  |  |  |  |  |  |  |
| Height | | A** | A** | 1 |  |  |  |  |  |  |  |  |
| Weight | | A** | A** | ** | 1 |  |  |  |  |  |  |  |
| Leaf_area | | A** | A** | ** | ** | 1 |  |  |  |  |  |  |
| Sugar | | A** | A** | ** | ** | ** | 1 |  |  |  |  |  |
| Protein | | * | * | A* | A** | A** | A** | 1 |  |  |  |  |
| Chlorophyll | | A** | A** | ** | ** | * | ** | ns | 1 |  |  |  |
| SOD | | ** | ** | A** | ns | ns | ns | ns | A* | 1 |  |  |
| POD | | ns | ns | ns | ns | ns | ns | ns | ns | ns | 1 |  |
| CAT | | A* | A* | ** | ** | ** | ** | ns | * | ns | ns | 1 |

**Table S3 The bioaccumulation factor (BAF) and translocation factor (TF) of oilseed rapes** (means±SD，n=6). Means within the same line that are followed by the different letter are significantly different at *P* < 0.05 based on one-way ANOVA.

|  | *B. napus* | | *B. juncea* | |
| --- | --- | --- | --- | --- |
|  | 10mg/kg | 30mg/kg | 10mg/kg | 30mg/kg |
| TF | 0.91±0.03a | 0.32±0.01c | 0.47±0.01b | 0.40±0.01d |
| BAF in leaf | 19.31±1.37a | 20.51±2.46a | 10.47±0.85b | 20.02±3.30a |
| BAF in root | 21.13±1.45c | 63.09±7.62a | 22.04±1.62c | 50.45±9.06b |

**Table S4 Pearson correlation of Cd concentration in soils of *B. napus* with rhizosphere physicochemical properties.**

| Pearson correlation | Soil_Cd | pH | TOC | TN | TP | NH_3_-N | NO_3_-N | AP | AK |
| --- | --- | --- | --- | --- | --- | --- | --- | --- | --- |
| Soil_Cd | 1 |  |  |  |  |  |  |  |  |
| pH | A** | 1 |  |  |  |  |  |  |  |
| TOC | A* | ns | 1 |  |  |  |  |  |  |
| TN | A** | ns | ns | 1 |  |  |  |  |  |
| TP | A* | ns | ns | ns | 1 |  |  |  |  |
| NH_3_-N | ns | ns | ns | ns | ns | 1 |  |  |  |
| NO_3_-N | A** | ns | ns | ** | * | ns | 1 |  |  |
| AP | ns | ns | ns | ns | ns | ns | ns | 1 |  |
| AK | ns | ns | ns | ns | ns | ns | ns | ns | 1 |

**Table S5 Pearson correlation of Cd concentration in soils of *B. juncea* with rhizosphere** **physicochemical properties.**

| Pearson correlation | Soil_Cd | pH | TOC | TN | TP | NH_3_-N | NO_3_-N | AP | | AK |
| --- | --- | --- | --- | --- | --- | --- | --- | --- | --- | --- |
| Soil_Cd | 1 |  |  |  |  |  |  | |  |  |
| pH | A** | 1 |  |  |  |  |  | |  |  |
| TOC | ns | ns | 1 |  |  |  |  | |  |  |
| TN | A** | ** | ns | 1 |  |  |  | |  |  |
| TP | ns | ns | A* | ns | 1 |  |  | |  |  |
| NH_3_-N | A* | ns | ns | ns | ns | 1 |  | |  |  |
| NO_3_-N | ns | ns | ns | ns | ns | ns | 1 | |  |  |
| AP | A* | ns | ns | * | ns | ns | ns | | 1 |  |
| AK | ns | ns | ns | ns | ns | ns | ns | | ns | 1 |

**Table S6 Pearson correlation of Cd concentration in soils of *B. napus* with bulk soil physicochemical properties.**

| Pearson correlation | Soil_Cd | pH | TOC | TN | TP | NH_3_-N | NO_3_-N | AP | AK |
| --- | --- | --- | --- | --- | --- | --- | --- | --- | --- |
| Soil_Cd | 1 |  |  |  |  |  |  |  |  |
| pH | A** | 1 |  |  |  |  |  |  |  |
| TOC | ns | ns | 1 |  |  |  |  |  |  |
| TN | A* | ** | ns | 1 |  |  |  |  |  |
| TP | A* | ** | ** | ** | 1 |  |  |  |  |
| NH_3_-N | ns | ns | ns | ns | ns | 1 |  |  |  |
| NO_3_-N | A* | * | ns | ** | ** | ns | 1 |  |  |
| AP | ns | ns | ns | ns | ns | ns | ns | 1 |  |
| AK | ns | ns | ns | ns | ns | ns | ns | ns | 1 |

**Table S7 Pearson correlation of Cd concentration in soils of *B. juncea* with bulk soil physicochemical properties.**

| Pearson correlation | Soil_Cd | pH | TOC | TN | TP | NH_3_-N | NO_3_-N | AP | AK |
| --- | --- | --- | --- | --- | --- | --- | --- | --- | --- |
| Soil_Cd | 1 |  |  |  |  |  |  |  |  |
| pH | A** | 1 |  |  |  |  |  |  |  |
| TOC | ns | ns | 1 |  |  |  |  |  |  |
| TN | A** | ns | ns | 1 |  |  |  |  |  |
| TP | ns | ns | ns | ** | 1 |  |  |  |  |
| NH_3_-N | A* | ns | ns | ** | ns | 1 |  |  |  |
| NO_3_-N | ns | ns | * | ** | ** | ns | 1 |  |  |
| AP | ns | ns | ns | ** | ** | ns | ** | 1 |  |
| AK | ** | ns | ns | ns | ns | ns | ns | ns | 1 |

**Table S8** **Pearson correlation of Cd in soils of *B. napus* with bacterial numbers.**

| Pearson correlation | Soil_Cd | Rhizosphere | Bulk soil |
| --- | --- | --- | --- |
| Soil_Cd | 1 |  |  |
| Rhizosphere | ns | 1 |  |
| Bulk soil | * | * | 1 |

**Table S9 Pearson correlation of Cd in soils of *B. juncea* with bacterial numbers.**

| Pearson correlation | Soil_Cd | Rhizosphere | Bulk soil |
| --- | --- | --- | --- |
| Soil_Cd | 1 |  |  |
| Rhizosphere | ns | 1 |  |
| Bulk soil | ns | ns | 1 |

**Table S10 Relationship between α-diversity of plant bacterial community in *B. napus* and environment variables.** Abbreviations: Yj: phyllosphere samples; Yn: endophytes samples from leaves; Gn: endophytes samples from roots, the same below.

| Samples | factor | Shannon | Simpson | Richness | Chao1 |
| --- | --- | --- | --- | --- | --- |
| Yj | Height | ns | ns | ns | ns |
|  | Weight | ns | ns | ns | ns |
|  | Leaf_area | ns | ns | ns | ns |
|  | Sugar | ns | ns | ns | ns |
|  | Protein | ns | ns | ns | ns |
|  | Chlorophyll | ns | ns | ns | ns |
|  | SOD | ns | ns | ns | ns |
|  | POD | ns | ns | ns | ns |
|  | CAT | ns | ns | ns | ns |
|  | Leaf_Cd | ns | ns | ns | ns |
| Yn | Height | ns | ns | ns | ns |
|  | Weight | ns | ns | ns | ns |
|  | Leaf_area | ns | ns | ns | ns |
|  | Sugar | ns | ns | ns | ns |
|  | Protein | ns | ns | ns | ns |
|  | Chlorophyll | ns | ns | ** | ns |
|  | SOD | ns | ns | ns | ns |
|  | POD | ns | ns | ns | ns |
|  | CAT | * | ns | ns | ns |
|  | Leaf_Cd | ns | ns | ns | ns |
| Gn | Height | ** | * | * | ns |
|  | Weight | * | ns | * | ns |
|  | Leaf_area | ** | * | * | ns |
|  | Sugar | ** | ns | * | ns |
|  | Protein | ns | ns | ns | ns |
|  | Chlorophyll | ns | * | ns | ns |
|  | SOD | A** | A* | A** | A* |
|  | POD | A* | ns | ns | ns |
|  | CAT | * | ns | ns | ns |
|  | Root_Cd | A** | A* | A* | ns |
|  | pH | * | * | * | * |
|  | TOC | * | * | * | * |
|  | TN | ns | * | ns | ns |
|  | TP | ns | ns | ns | ns |
|  | NH_3_-N | ns | ns | ns | ns |
|  | NO_3_-N | ns | ns | ns | ns |
|  | AP | ns | ns | ns | ns |
|  | AK | ns | ns | ns | ns |

**Table S11 Relationship between α-diversity of plant bacterial community in *B. juncea* and environment variables.**

| Samples | factor | Shannon | Simpson | Richness | Chao1 |
| --- | --- | --- | --- | --- | --- |
| Yj | Height | ns | ns | ns | * |
|  | Weight | ns | ns | ns | ns |
|  | Leaf_area | ns | ns | ns | ns |
|  | Sugar | ns | ns | * | * |
|  | Protein | ns | ns | ns | ns |
|  | Chlorophyll | ns | ns | * | * |
|  | SOD | ns | ns | ns | ns |
|  | POD | ns | ns | ns | ns |
|  | CAT | ns | ns | * | * |
|  | Leaf_Cd | ns | ns | ns | ns |
| Yn | Height | ns | ns | ns | ns |
|  | Weight | ns | ns | ns | ns |
|  | Leaf_area | ns | ns | ns | ns |
|  | Sugar | ns | ns | ns | ns |
|  | Protein | ns | ns | ns | ns |
|  | Chlorophyll | ns | ns | ns | ns |
|  | SOD | ns | ns | ns | ns |
|  | POD | ns | A* | ns | ns |
|  | CAT | ns | n | ns | ns |
|  | Leaf_Cd | ns | ns | ns | ns |
| Gn | Height | ns | ns | ns | ns |
|  | Weight | ns | ns | ns | ns |
|  | Leaf_area | ns | ns | ns | ns |
|  | Sugar | ns | ns | ns | ns |
|  | Protein | ns | ns | ns | ns |
|  | Chlorophyll | ns | ns | ns | ns |
|  | SOD | ns | ns | ns | ns |
|  | POD | ns | ns | ns | ns |
|  | CAT | ns | ns | ns | ns |
|  | Root_Cd | ns | ns | ns | ns |
|  | pH | ns | ns | ns | ns |
|  | TOC | A* | ns | ns | ns |
|  | TN | ns | ns | ns | ns |
|  | TP | ns | ns | ns | ns |
|  | NH_3_-N | ns | ns | ns | ns |
|  | NO_3_-N | ns | ns | ns | ns |
|  | AP | ns | ns | ns | ns |
|  | AK | ns | ns | ns | ns |

**Table S12 Relationship between α-diversity of soil bacterial community in *B. napus* and environment variables.** Abbreviations:R: rhizosphere soil samples; NR: bulk soil samples, the same below.

| Samples | factor | Shannon | Simpson | Richness | Chao1 |
| --- | --- | --- | --- | --- | --- |
| R | Height | ** | ** | ** | ns |
|  | Weight | ** | ** | * | ns |
|  | Leaf_area | ** | ** | ** | ns |
|  | Sugar | ** | * | * | ns |
|  | Protein | ns | ns | ns | A* |
|  | Chlorophyll | ns | ns | ns | ns |
|  | SOD | A** | A** | A** | ns |
|  | POD | ns | ns | ns | ns |
|  | CAT | ** | * | * | ns |
|  | Soil_Cd | A** | A** | A** | ns |
|  | pH | * | * | * | ns |
|  | TOC | * | ns | ns | ns |
|  | TN | * | * | ns | ns |
|  | TP | * | ns | ns | ns |
|  | NH_3_-N | ns | ns | ns | ns |
|  | NO_3_-N | ** | ** | * | ns |
|  | AP | ns | ns | ns | ns |
|  | AK | ns | ns | ns | ns |
| NR | pH | ns | ns | ns | ns |
|  | TOC | ns | ns | ns | ns |
|  | TN | ns | * | ns | ns |
|  | TP | ns | ns | ns | ns |
|  | NH_3_-N | ns | ns | ns | ns |
|  | NO_3_-N | * | * | ns | ns |
|  | AP | ns | ns | ns | ns |
|  | AK | ns | ns | ns | ns |
|  | Soil_Cd | A* | A* | ns | ns |

**Table S13 Relationship between α-diversity of soil bacterial community in *B. juncea* and environment variables.**

| Samples | factor | Shannon | Simpson | Richness | Chao1 |
| --- | --- | --- | --- | --- | --- |
| R | Height | ns | ** | ns | ns |
|  | Weight | * | ** | ns | ns |
|  | Leaf_area | ns | * | ns | ns |
|  | Sugar | ns | * | ns | ns |
|  | Protein | A* | A** | ns | A* |
|  | Chlorophyll | ns | * | ns | ns |
|  | SOD | A* | A* | ns | ns |
|  | POD | ns | ns | ns | ns |
|  | CAT | ns | ns | ns | ns |
|  | Soil_Cd | A* | A** | ns | ns |
|  | pH | ns | ns | ns | ns |
|  | TOC | ns | ns | ns | ns |
|  | TN | ns | ns | ns | ns |
|  | TP | ns | ns | ns | ns |
|  | NH_3_-N | ns | ns | ns | ns |
|  | NO_3_-N | ns | ns | ns | ns |
|  | AP | ns | ns | ns | ns |
|  | AK | ns | ns | ns | ns |
| NR | pH | ** | * | ** | ** |
|  | TOC | ns | ns | ns | ns |
|  | TN | ns | ns | ns | ns |
|  | TP | ns | ns | ns | ns |
|  | NH_3_-N | ns | ns | ns | ns |
|  | NO_3_-N | ns | ns | ns | ns |
|  | AP | ns | ns | ns | ns |
|  | AK | A* | A** | A** | A* |
|  | Soil_Cd | A* | A* | A* | A** |

**Table S14 Dissimilarity tests of bacterial communities in plant and soil in *B. napus* from two different group types based on Jaccard distance method.** Abbreviations: Yj, phyllosphere samples; Yn, endophytes samples from leaves; Gn, endophytes samples from roots; R, rhizosphere soil samples; NR, bulk soil samples; CK, treatment with 0 mg/kg Cd; C10, treatment with 10 mg/kg Cd; C30, treatment with 30 mg/kg Cd, the same below.

| Groups | | MRPP | | ANOSIM | | PERMANOVA | |
| --- | --- | --- | --- | --- | --- | --- | --- |
|  |  | *δ* | *P* | *R* | *P* | *R* | *P* |
| Plant | Yj | 0.684 | 0.183 | 0.072 | 0.085 | 1.044 | 0.205 |
|  | Yn | 0.727 | 0.202 | 0.031 | 0.324 | 1.054 | 0.231 |
|  | Gn | 0.660 | 0.010 | 0.179 | 0.006 | 1.595 | 0.013 |
|  | CK | 0.690 | 0.001 | 0.750 | 0.001 | 3.755 | 0.001 |
|  | C10 | 0.680 | 0.001 | 0.929 | 0.001 | 4.144 | 0.001 |
|  | C30 | 0.702 | 0.001 | 0.819 | 0.001 | 3.690 | 0.001 |
| Soil | R | 0.468 | 0.003 | 0.317 | 0.001 | 1.298 | 0.003 |
|  | NR | 0.453 | 0.001 | 0.378 | 0.001 | 1.397 | 0.001 |
|  | CK | 0.456 | 0.009 | 0.380 | 0.005 | 1.334 | 0.016 |
|  | C10 | 0.477 | 0.010 | 0.244 | 0.005 | 1.341 | 0.014 |
|  | C30 | 0.449 | 0.003 | 0.865 | 0.005 | 1.762 | 0.004 |

**Table S15 Dissimilarity tests of bacterial communities in plant and soil in *B. juncea* from two different group types** **based on Jaccard distance method.**

| Groups | | MRPP | | ANOSIM | | PERMANOVA | |
| --- | --- | --- | --- | --- | --- | --- | --- |
|  |  | *δ* | *P* | *R* | *P* | *R* | *P* |
| Plant | Yj | 0.658 | 0.069 | 0.103 | 0.052 | 1.127 | 0.051 |
|  | Yn | 0.760 | 0.093 | 0.107 | 0.077 | 1.107 | 0.094 |
|  | Gn | 0.640 | 0.024 | 0.123 | 0.046 | 1.139 | 0.021 |
|  | CK | 0.673 | 0.001 | 0.845 | 0.001 | 4.473 | 0.001 |
|  | C10 | 0.703 | 0.001 | 0.800 | 0.001 | 3.680 | 0.001 |
|  | C30 | 0.683 | 0.001 | 0.785 | 0.001 | 3.842 | 0.001 |
| Soil | R | 0.463 | 0.001 | 0.409 | 0.001 | 1.434 | 0.001 |
|  | NR | 0.457 | 0.007 | 0.180 | 0.006 | 1.169 | 0.008 |
|  | CK | 0.452 | 0.027 | 0.159 | 0.036 | 1.123 | 0.026 |
|  | C10 | 0.469 | 0.012 | 0.154 | 0.042 | 1.254 | 0.020 |
|  | C30 | 0.460 | 0.001 | 0.470 | 0.003 | 1.464 | 0.003 |

**Table S16 Mantel analysis of the relationship between the *B. napus*’s bacterial community structure and environmental variables based on Bray Curtis (BC) and Jaccard (JC) method.**

| Samples | Environmental factors | *r*.BC | *p*.BC | *r*.JC | *p*.JC |
| --- | --- | --- | --- | --- | --- |
| Yj | Height | 0.002 | 0.468 | -0.053 | 0.595 |
|  | Weight | 0.088 | 0.138 | 0.038 | 0.398 |
|  | Leaf_area | 0.024 | 0.363 | -0.046 | 0.618 |
|  | Sugar | -0.041 | 0.568 | -0.090 | 0.672 |
|  | Protein | -0.023 | 0.466 | -0.041 | 0.529 |
|  | Chlorophyll | 0.250 | 0.105 | 0.516 | 0.014 |
|  | SOD | -0.028 | 0.541 | -0.126 | 0.944 |
|  | POD | -0.011 | 0.460 | -0.050 | 0.561 |
|  | CAT | -0.099 | 0.737 | -0.103 | 0.731 |
|  | Leaf_Cd | 0.024 | 0.387 | -0.046 | 0.615 |
| Yn | Height | -0.120 | 0.936 | 0.094 | 0.148 |
|  | Weight | -0.107 | 0.922 | 0.206 | 0.013 |
|  | Leaf_area | -0.039 | 0.632 | 0.197 | 0.010 |
|  | Sugar | -0.114 | 0.829 | 0.152 | 0.113 |
|  | Protein | 0.030 | 0.363 | 0.069 | 0.256 |
|  | Chlorophyll | 0.025 | 0.419 | 0.163 | 0.149 |
|  | SOD | -0.118 | 0.881 | -0.049 | 0.635 |
|  | POD | 0.132 | 0.113 | -0.212 | 0.991 |
|  | CAT | -0.012 | 0.502 | -0.084 | 0.738 |
|  | Leaf_Cd | -0.124 | 0.941 | 0.107 | 0.123 |
| Gn | Height | 0.362 | 0.012 | 0.264 | 0.020 |
|  | Weight | 0.276 | 0.010 | 0.179 | 0.021 |
|  | Leaf_area | 0.276 | 0.015 | 0.218 | 0.015 |
|  | pH | 0.200 | 0.059 | 0.139 | 0.186 |
|  | TOC | 0.261 | 0.046 | 0.090 | 0.240 |
|  | TN | -0.059 | 0.614 | -0.145 | 0.842 |
|  | TP | 0.038 | 0.314 | -0.060 | 0.547 |
|  | NH_3_-N | 0.093 | 0.213 | 0.134 | 0.188 |
|  | NO_3_-N | 0.025 | 0.342 | 0.027 | 0.360 |
|  | AP | 0.050 | 0.232 | -0.039 | 0.525 |
|  | AK | -0.089 | 0.741 | -0.127 | 0.725 |
|  | Root_Cd | 0.398 | 0.011 | 0.302 | 0.016 |

**Table S17 Mantel analysis of the relationship between the *B. juncea*’s bacterial community structure and environmental variables based on Bray Curtis (BC) and Jaccard (JC) method.**

| Samples | Environmental factors | *r*.BC | *p*.BC | *r*.JC | *p*.JC |
| --- | --- | --- | --- | --- | --- |
| Yj | Height | 0.095 | 0.136 | 0.041 | 0.322 |
|  | Weight | 0.020 | 0.359 | 0.001 | 0.471 |
|  | Leaf_area | 0.004 | 0.419 | 0.006 | 0.433 |
|  | Sugar | -0.007 | 0.475 | 0.022 | 0.374 |
|  | Protein | 0.051 | 0.370 | -0.077 | 0.652 |
|  | Chlorophyll | -0.008 | 0.509 | 0.093 | 0.098 |
|  | SOD | -0.129 | 0.817 | -0.250 | 0.987 |
|  | POD | 0.204 | 0.058 | 0.130 | 0.149 |
|  | CAT | -0.004 | 0.508 | 0.035 | 0.358 |
|  | Leaf_Cd | 0.072 | 0.213 | 0.007 | 0.425 |
| Yn | Height | -0.136 | 0.966 | -0.128 | 0.939 |
|  | Weight | -0.205 | 1.000 | -0.060 | 0.752 |
|  | Leaf_area | -0.088 | 0.829 | 0.101 | 0.139 |
|  | Sugar | -0.162 | 0.951 | 0.005 | 0.507 |
|  | Protein | -0.048 | 0.521 | -0.094 | 0.722 |
|  | Chlorophyll | -0.131 | 0.99 | 0.008 | 0.430 |
|  | SOD | -0.093 | 0.642 | -0.035 | 0.575 |
|  | POD | 0.039 | 0.35 | -0.132 | 0.841 |
|  | CAT | -0.141 | 0.806 | 0.049 | 0.346 |
|  | Leaf_Cd | -0.174 | 0.996 | -0.121 | 0.883 |
| Gn | Height | 0.008 | 0.428 | 0.018 | 0.392 |
|  | Weight | 0.053 | 0.259 | 0.047 | 0.284 |
|  | Leaf_area | 0.154 | 0.069 | 0.101 | 0.125 |
|  | pH | 0.066 | 0.239 | 0.093 | 0.171 |
|  | TOC | 0.170 | 0.114 | -0.057 | 0.617 |
|  | TN | 0.057 | 0.292 | 0.114 | 0.174 |
|  | TP | 0.206 | 0.084 | 0.120 | 0.187 |
|  | NH_3_-N | -0.042 | 0.602 | 0.106 | 0.242 |
|  | NO_3_-N | -0.179 | 0.945 | -0.246 | 0.986 |
|  | AP | 0.057 | 0.340 | 0.010 | 0.432 |
|  | AK | 0.069 | 0.241 | -0.083 | 0.759 |
|  | Root_Cd | -0.042 | 0.663 | 0.053 | 0.281 |

**Table S18 Mantel analysis of the relationship between the soil bacterial community structure in *B. juncea* and environmental variables** **based on Bray Curtis (BC) and Jaccard (JC) method.**

| Samples | Environmental factors | *r*.BC | *p*.BC | *r*.JC | *p*.JC |
| --- | --- | --- | --- | --- | --- |
| Rhizosphere soil | Height | 0.179 | 0.026 | 0.092 | 0.170 |
|  | Weight | 0.235 | 0.010 | 0.117 | 0.090 |
|  | Leaf_area | 0.240 | 0.011 | 0.223 | 0.026 |
|  | pH | 0.078 | 0.239 | 0.094 | 0.179 |
|  | TOC | -0.011 | 0.495 | -0.015 | 0.491 |
|  | TN | 0.092 | 0.256 | 0.270 | 0.034 |
|  | TP | 0.104 | 0.249 | 0.137 | 0.179 |
|  | NH_3_-N | -0.044 | 0.587 | 0.241 | 0.075 |
|  | NO_3_-N | 0.034 | 0.360 | -0.103 | 0.741 |
|  | AP | 0.058 | 0.368 | 0.109 | 0.225 |
|  | AK | -0.117 | 0.831 | -0.123 | 0.809 |
|  | Soil_Cd | 0.232 | 0.023 | 0.102 | 0.183 |
| Bulk soil | pH | 0.407 | 0.001 | 0.461 | 0.001 |
|  | TOC | 0.033 | 0.380 | 0.039 | 0.369 |
|  | TN | 0.007 | 0.429 | 0.035 | 0.397 |
|  | TP | -0.187 | 0.917 | -0.247 | 0.959 |
|  | NH_3_-N | 0.045 | 0.348 | 0.062 | 0.324 |
|  | NO_3_-N | -0.087 | 0.674 | -0.092 | 0.699 |
|  | AP | -0.001 | 0.429 | -0.047 | 0.590 |
|  | AK | 0.101 | 0.208 | 0.145 | 0.134 |
|  | Soil_Cd | 0.445 | 0.005 | 0.511 | 0.001 |

**
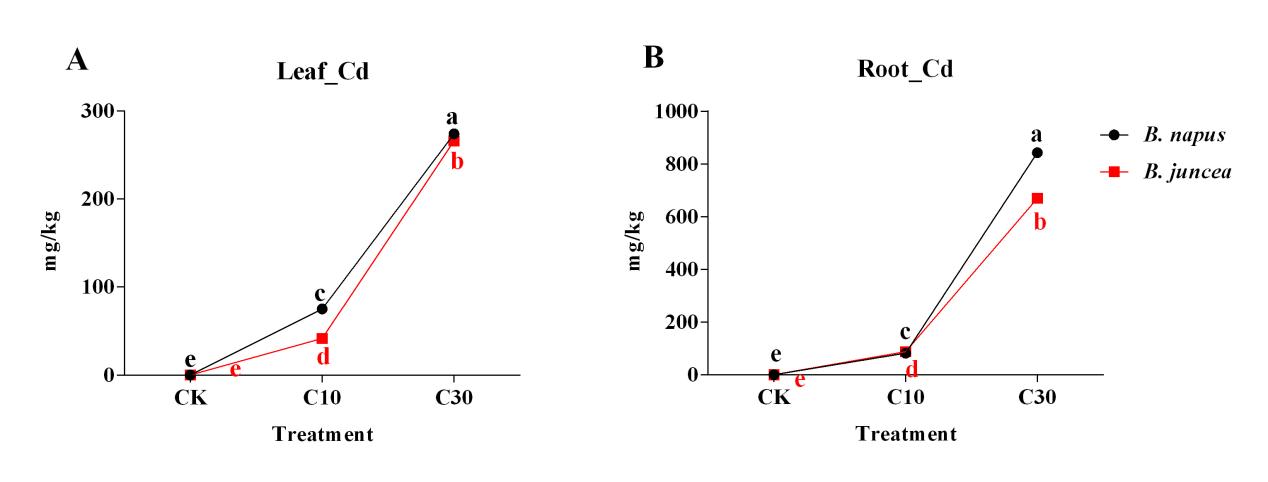
**

**Figure S1 The accumulation of Cd in plant leaves (A) and roots (B).** Error bars show standard deviation (n = 6). Means with different lowercase letters are significantly different at *P* < 0.05 based on one-way ANOVA, the same below. Abbreviations: CK, treatment with 0 mg/kg Cd; C10, treatment with 10 mg/kg Cd; C30, treatment with 30 mg/kg Cd, the same below.


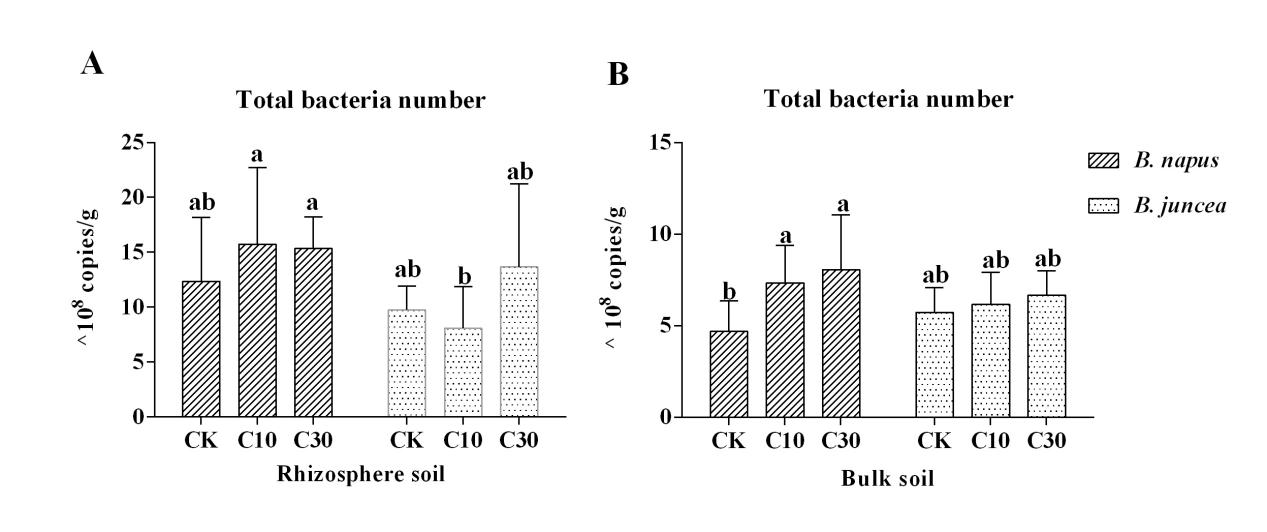


**Figure S2 Variations of total bacteria copies in Cd contaminated soils of rhizosphere (A) and bulk soil (B).**


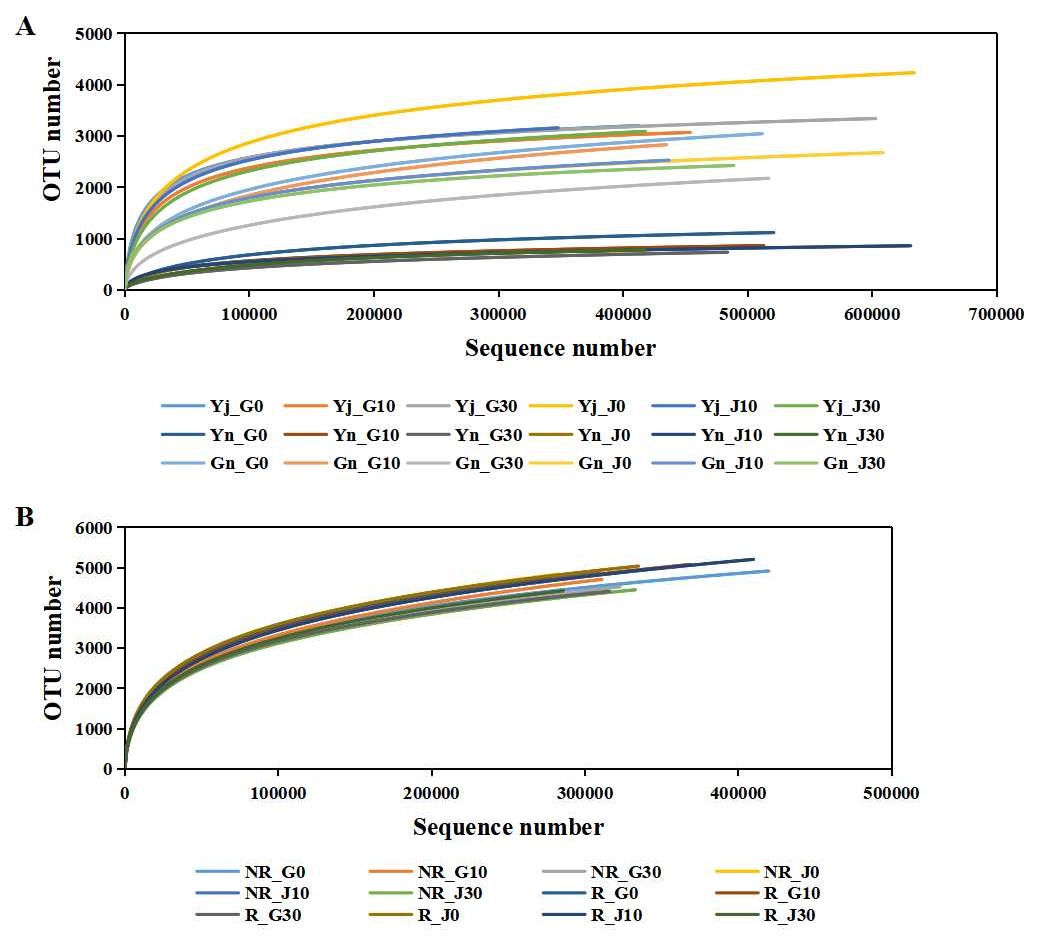


**Figure S3 Rarefaction curve of the bacterial community in plants (A) and soils (B).** Abbreviations: G0/J0, treatment with 0mg/kg Cd in *B. napus* /*B. juncea*; G10/J10, treatment with 10mg/kg Cd in *B. napus* */B. juncea*; G30/J30, treatment with 30mg/kg Cd in *B. napus* /*B. juncea* ; Yj, phyllosphere samples; Yn, endophytes samples from leaves; Gn, endophytes samples from roots; R, rhizosphere soil samples; NR, bulk soil samples, the same below.


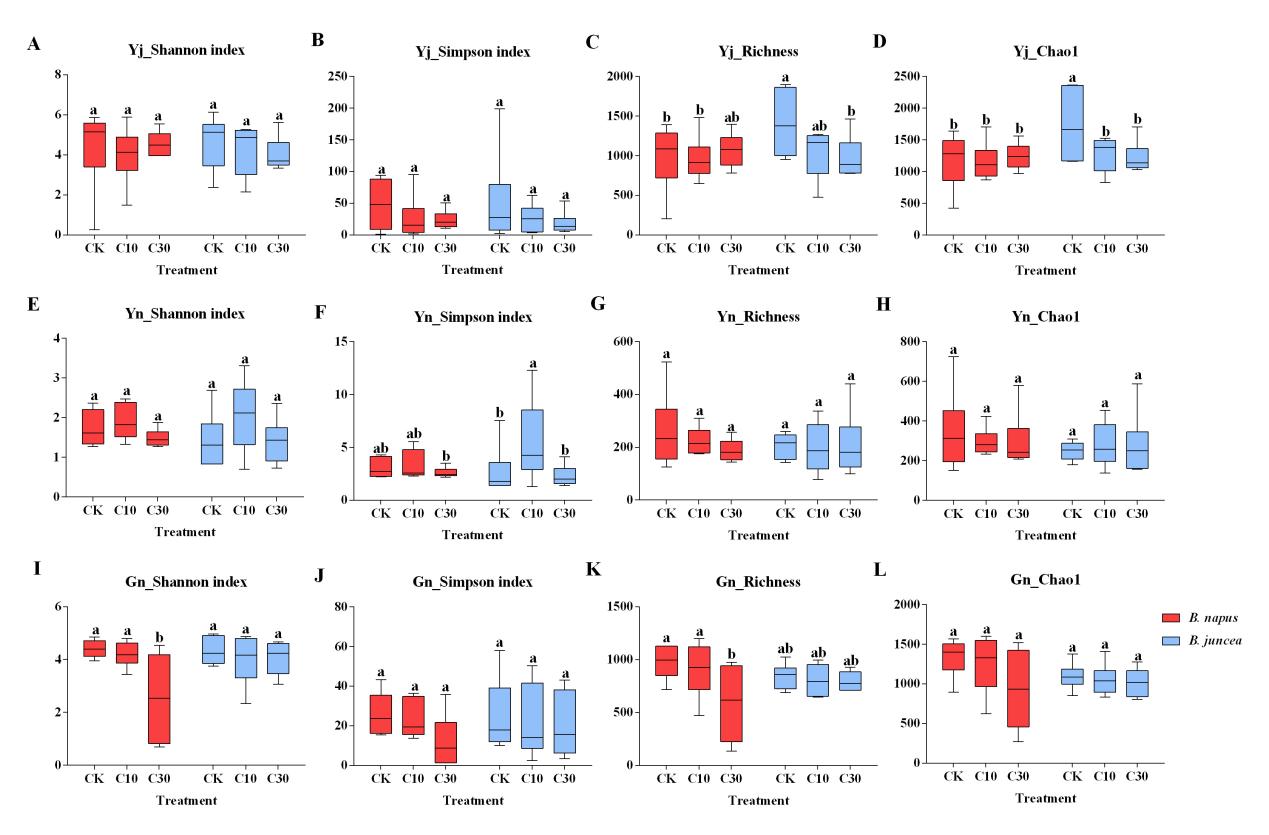


**Figure S4 The effect of Cd on α-diversity of plant bacterial communities.** Error bars show standard deviation (n = 6). Means with different lowercase letters are significantly different at *P* < 0.05 based on one-way ANOVA. Abbreviations: Yj, phyllosphere samples (A-D); Yn, endophytes samples from leaves (E-H); Gn, endophytes samples from roots (I-L).


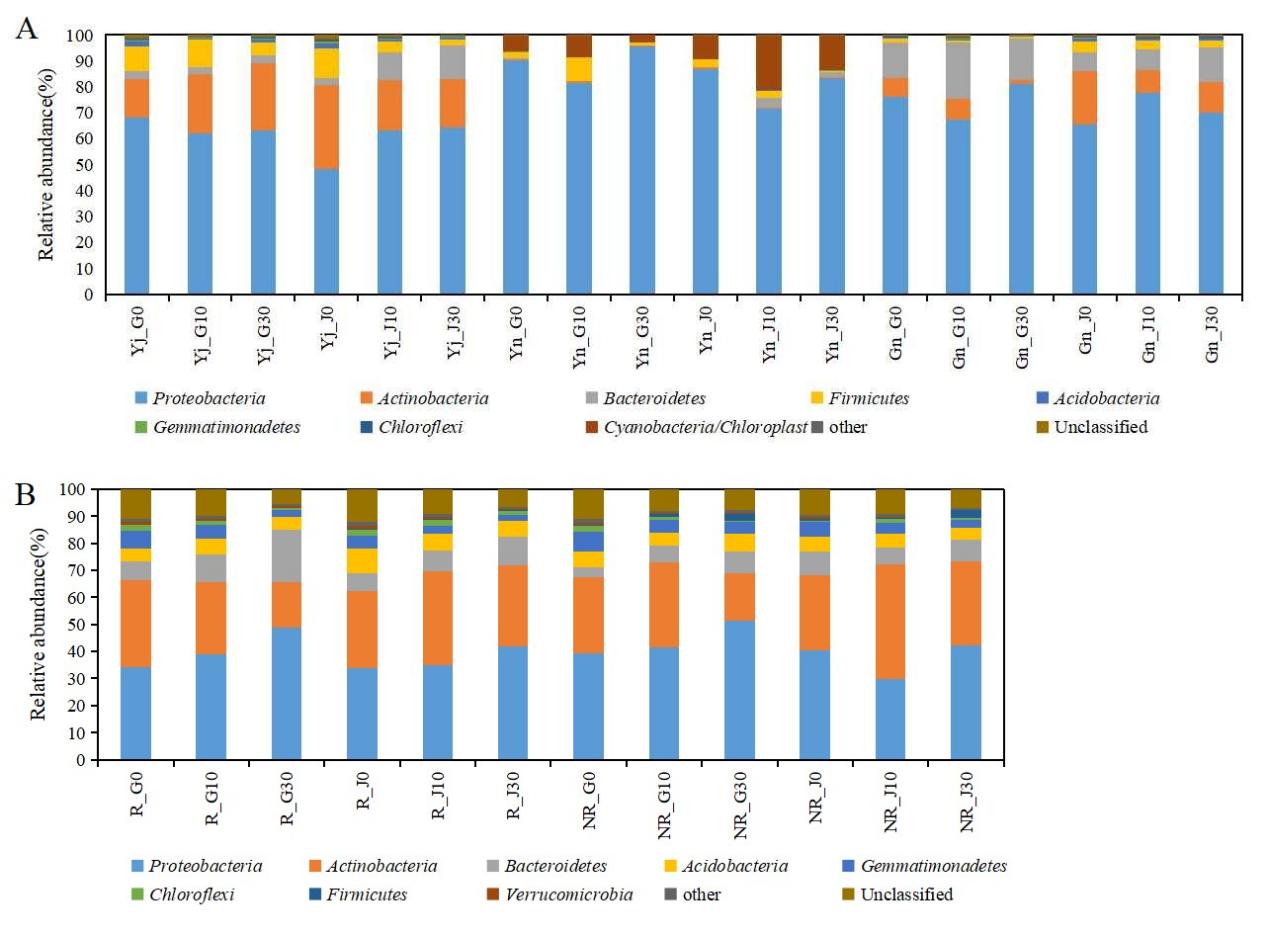


**Figure S5 Relative abundance of the top 10 phyla in plant (A) and soil (B) associated bacterial communities**


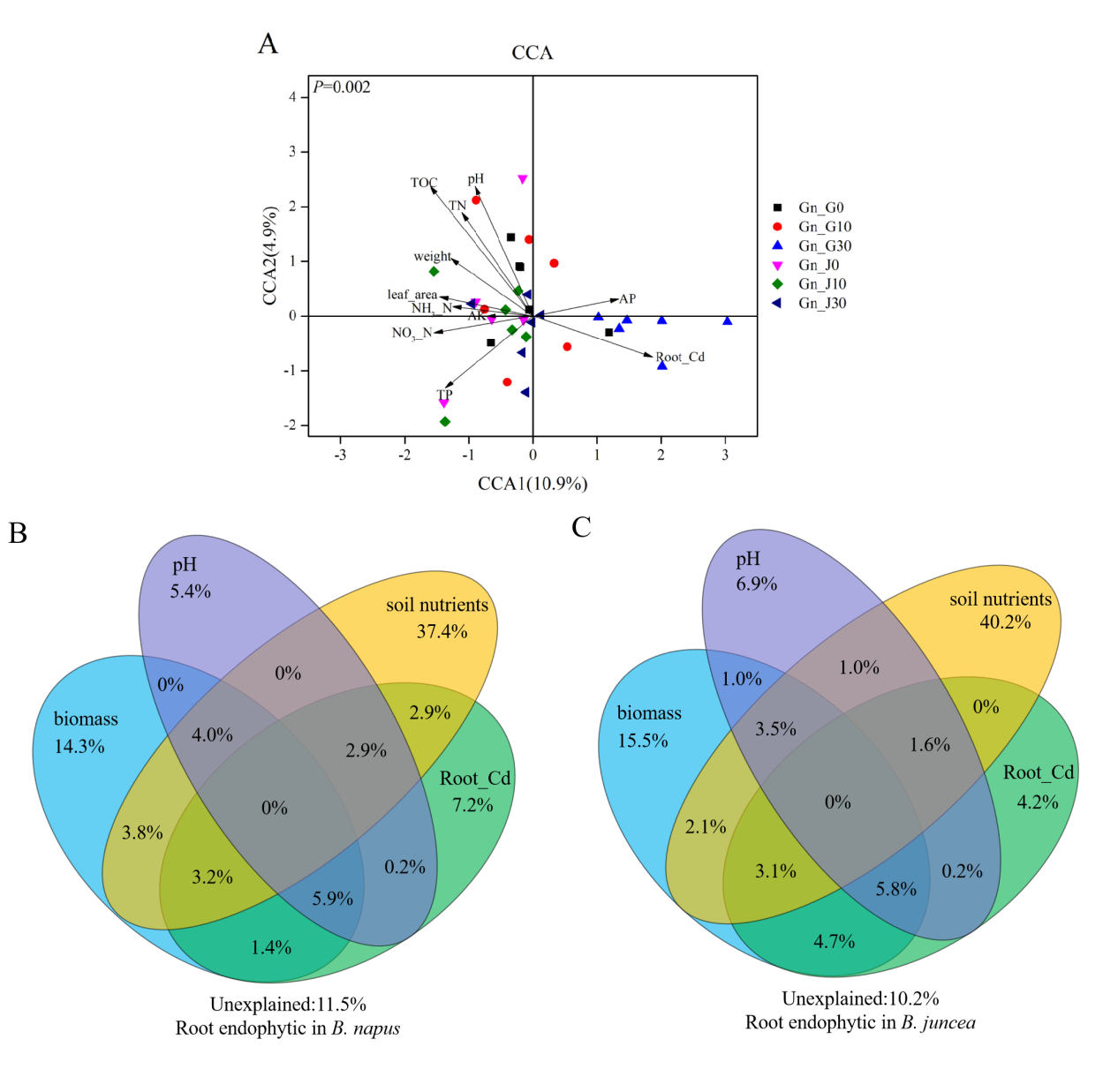


**Figure S6 Canonical correspondence analysis (CCA) on root endophytic bacterial communities with the environmental variables (A) and CCA-based variation partitioning analysis (VPA) of bacterial communities explained by environmental variables (B&C).**


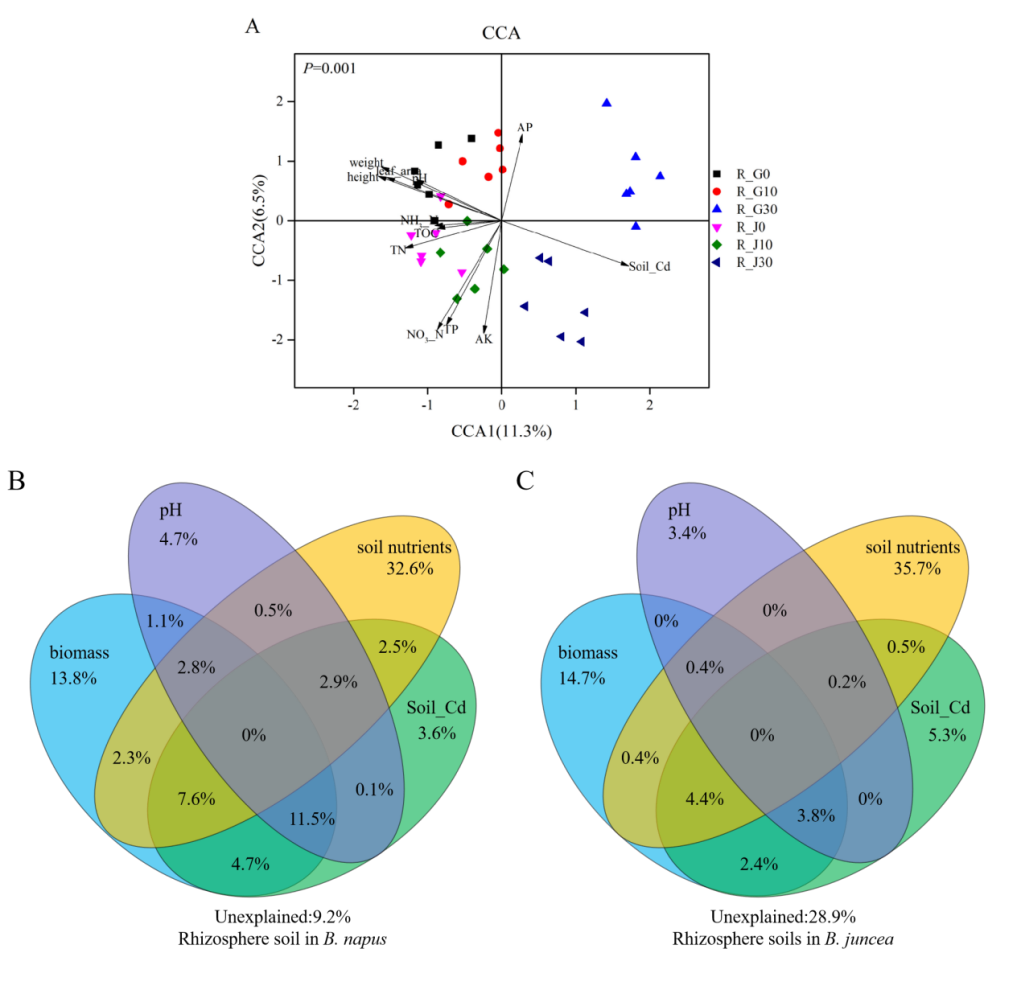


**Figure S7 Canonical correspondence analysis (CCA) on rhizosphere soil bacterial communities with the environmental variables (A) and CCA-based variation partitioning analysis (VPA) of bacterial communities explained by environmental variables (B&C).**
